# European Sitting Championship: Prevalence and Correlates of Self-Reported Sitting Time in the 28 European Union Member States

**DOI:** 10.1371/journal.pone.0149320

**Published:** 2016-03-02

**Authors:** Anne Loyen, Hidde P. van der Ploeg, Adrian Bauman, Johannes Brug, Jeroen Lakerveld

**Affiliations:** 1 Department of Epidemiology and Biostatistics, VU University Medical Center, EMGO Institute for Health and Care Research, Amsterdam, The Netherlands; 2 Department of Public and Occupational Health, VU University Medical Center, EMGO Institute for Health and Care Research, Amsterdam, The Netherlands; 3 Prevention Research Collaboration, School of Public Health, University of Sydney, Sydney, Australia; Vanderbilt University, UNITED STATES

## Abstract

**Objective:**

Sedentary behaviour is increasingly recognized as an important health risk, but comparable data across Europe are scarce. The objective of this study was to explore the prevalence and correlates of self-reported sitting time in adults across and within the 28 European Union Member States.

**Methods:**

This study reports data from the Special Eurobarometer 412. In 2013, 27,919 randomly selected Europeans (approximately 1000 per Member State) were interviewed face-to-face. Sitting time on a usual day was self-reported and dichotomised into sitting less- and more than 7.5 hours per day. Uni- and multivariate odds ratios of sitting more than 7.5 hours per day were assessed by country and socio-demographic variables using binary logistic regression analyses. The analyses were stratified by country to study the socio-demographic correlates of sitting time within the different countries.

**Results:**

A total of 26,617 respondents were included in the analyses. Median sitting time was five hours per day. Across Europe, 18.5 percent of the respondents reported to sit more than 7.5 hours per day, with substantial variation between countries (ranging from 8.9 to 32.1 percent). In general, northern European countries reported more sitting than countries in the south of Europe. ‘Current occupation’ and ‘age when stopped education’ were found to be the strongest correlates of sitting time, both across Europe and within most Member States. Compared to manual workers, the odds ratio of sitting more than 7.5 hours per day was 5.00 for people with white collar occupations, 3.84 for students, and 3.65 for managers.

**Conclusions:**

There is substantial variation in self-reported sitting time among European adults across countries as well as socio-demographic groups. While regular surveillance of (objectively measured) sedentary behaviour is needed, the results of this study provide entry points for developing targeted interventions aimed at highly sedentary populations, such as people with sedentary occupations.

## Introduction

Sedentary behaviour (SB) is increasingly recognized as an important health risk. SB is often quantified as sitting time and defined as any waking behaviour characterized by an energy expenditure ≤1.5 metabolic equivalents (METs) while in a sitting or reclining position.[1] SB is different from physical inactivity, which indicates not meeting physical activity (PA) recommendations.[2] Recent reviews and meta-analyses have linked adult SB to type 2 diabetes,[3–6] cardiovascular diseases,[3,5,7] cardiovascular mortality[3–5,7,8] and all-cause mortality.[3–11] Moreover, it is suggested that these risks of SB may be partly independent of PA levels.[1]

Research into the prevalence and determinants of SB is essential to monitor population levels, identify populations with high levels of SB, and plan targeted actions for those populations. Moreover, cross-country monitoring allows comparison and benchmarking. However, comparable SB data across countries are scarce.[12,13] In Europe, the most comprehensive cross-national population data source on SB currently available are the Eurobarometer surveys.[14] While Eurobarometers are typically focussed on public opinion issues relating to the European Union (EU), questions about the respondents’ SB (in the form of self-reported sitting time) are included in some surveys.

SB was assessed by Eurobarometer surveys in 2002, 2005 and 2013 [15–17]. While the same root question was used in all three surveys, the response scale was different in the 2013 survey. In an examination of the trends of sitting time across these surveys, Milton and colleagues[18] concluded that mean(SD) sitting time declined from 316(179) minutes per day in 2002 to 292(138) minutes per day in 2013. The prevalence of high sitting (defined as sitting more than 7.5 hours/day) showed the same trend from 23.1 percent in 2002 to 17.8 percent in 2013. In both the 2002 and 2005 Eurobarometer samples, males, younger people and higher educated people reported more sitting[19,20].

The current study focusses on the 2013 Eurobarometer data. This survey included multiple socio-demographic characteristics, including characteristics that were not included in previous Eurobarometer surveys. It therefore provided the opportunity to study these characteristics as correlates of sitting too much in multivariate models. In addition, the relatively large sample and high number of observations per country allowed us to examine within-country associations. The objective of this study is therefore to explore the prevalence and a wide range of socio-demographic correlates of self-reported sitting time in adults across and within the 28 EU Member States surveyed in the 2013 Eurobarometer.

## Materials and Methods

### Eurobarometer

**Fig 1:**
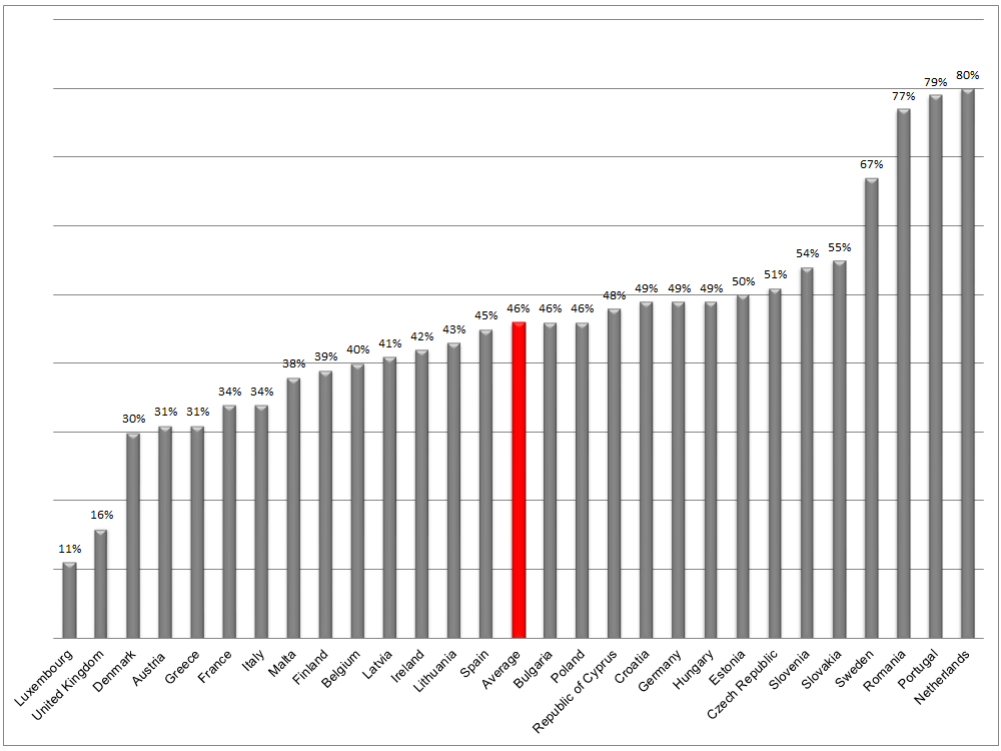
The response rate of the Eurobarometer survey per country.

We used data from the Special Eurobarometer 412 “Sport and physical activity”, which was carried out in the 28 EU Member States in November and December 2013 by the market research company TNS opinion & social[17]. Eurobarometers are biannual, cross-sectional surveys conducted on behalf of the European Commission that cover approximately 1000 respondents per EU Member State. Participants were sampled using a multistage random sampling design, with multiple sampling points based on country specific population size and -density. Households were selected by starting at a random address and following a pre-defined random route. In each household, one respondent was selected based on the closest birthday to the date of the interview. Data was obtained from people aged 15 years and over by the Eurobarometer, but in our research we only used data from people aged 18 years and over. Participants were interviewed face-to-face at home in their mother tongue using a standardized protocol. A total of 27,919 Europeans were surveyed, ranging from 500 in the Republic of Cyprus and Malta to 1600 in Germany. The average response rate was 46 percent, ranging from 11 percent in Luxembourg to 80 percent in the Netherlands. The response rates per country are presented in Fig 1. The European Commission approved the study protocols and informed consent was obtained from all participants. The information was anonymized and de-identified prior to analysis.

### Variables

#### Sitting time

Sitting time was measured with a variant of the validated International Physical Activity Questionnaire (IPAQ[21,22])-short sitting item: “How much time do you spend sitting on a usual day? This may include time spent at a desk, visiting friends, studying or watching television.” There were eleven response options: 1 hour or less, 1 hour to 1.5 hour, 1.5 hour to 2.5 hours—7.5 hours to 8.5 hours (in one-hour intervals), more than 8.5 hours and ‘don’t know’. In order to estimate median sitting time we transformed the sitting variable by assigning the midpoint to each category (e.g. the ‘1.5 hours to 2.5 hours’ category was transformed into ‘120 minutes’). In addition, we dichotomized the sitting variable into sitting ≤7.5 hours per day and sitting >7.5 hours per day to study high versus low sitting. This cut-point was based on a recent meta-analysis, which suggested that the risk of all-cause mortality increases if adults sit more than approximately 7 hours per day.[9]

#### Socio-demographic variables

All variables were self-reported during a face-to-face interview in which the interviewer read out the questions and noted the answers. Country was indicated by the interviewer; we recoded West- and East-Germany into Germany and England and Northern Ireland into United Kingdom. Age was recoded into six categories: 15–24 years, 25–34 years, 35–44 years, 45–54 years, 55–64 years, 65 years and over. Marital status was recoded into four categories: unmarried, (re-) married or with partner, divorced or separated, widowed. Level of education was assessed by the question “How old were you when you stopped full-time education?” and was recoded into four categories: up to 15 years, 16–19 years, 20 years or older, still studying. Current occupation was recoded into seven categories: self-employed (farmer/fisherman, professional, owner of a shop, craftsmen, business proprietors), managers (employed professional, general management, middle management), other white collars (employed position at desk, employed position travelling), manual worker (employed position service job, supervisor, skilled manual worker, unskilled manual worker), house persons, unemployed, retired, students. PA was assessed by the IPAQ-short, asking about the number of days and the average time respondents participated in vigorous PA, moderate PA, and walking in the last seven days. Response options were: 30 minutes or less, 31 to 60 minutes, 61 to 90 minutes, 91 to 120 minutes, more than 120 minutes, never, and don’t know. The width of these categories made it impossible to assess whether respondents met PA recommendations. Alternatively, we calculated MET-minutes/week using the following formula: (days of vigorous PA * time in vigorous PA * 8.0) + (days of moderate PA * time in moderate PA * 4.0) + (days walking * time walking * 3.3).[21] In this process, we took the midpoint of each category to represent the time (e.g. the ‘31 to 60 minutes’ category was transformed into ‘45 minutes’) and capped the ‘more than 120 minutes’ category at 135 minutes. Subsequently, we constructed four categories based on quartiles: least active quartile, second quartile, third quartile, most active quartile. Type of community was self-reported in three categories: rural area or village, small or medium sized town, large town. We computed the variable ‘Children aged <15 years living in the household’ by summing up the number of children aged <10 years and those aged 10 to 14 years and coded it into four categories: none, one, two, three or more. Television-, computer-, and car ownership were dichotomous variables, i.e. yes or no. We recoded Internet use frequency into three categories: never/no access, often/sometimes, every day. Difficulties paying bills was measured in three categories: almost never/never, from time to time, most of the time. Finally, life satisfaction was measured with four categories: “On the whole, are you very satisfied, fairly satisfied, not very satisfied, or not at all satisfied with the life you lead?”

### Statistical analysis

No sampling weights were applied in the analyses. We excluded participants aged <18 years because our focus is on adults, as well as participants who answered ‘don’t know’ on the sitting question since this is our main outcome. For all other variables, the response options ‘refusal’ and ‘don’t know’ were indicated as missing values. Sample characteristics and sitting prevalence were analysed with descriptive statistics. Univariate odds ratios (ORs) of sitting >7.5 hours per day were assessed by country and socio-demographic variables using binary logistic regression analyses with dummy variables where appropriate. For the ordinal variables, we also assessed the overall trends.

We wanted to compare each country with all other countries, instead of choosing one country as a reference. Therefore, the multivariate ORs were assessed in 29 separate logistic regression analyses; 28 analyses to assess the ORs of the 28 countries, and one additional analysis to assess the ORs of all socio-demographic variables. A country-specific variable was computed for each country to distinguish people living in that country from people living in all other countries. To assess the OR of each country, the analysis included the appropriate country-specific variable and all socio-demographic variables. To assess the ORs of all socio-demographic characteristics, the final analysis included all country-specific variables as well as all socio-demographic variables.

Because of co-linearity between the education and occupation variables (i.e. the group ‘students’ is identified in both variables) we constructed and reported two multivariate models: one including ‘Age when stopped full-time education’ and excluding ‘Current occupation’, and vice versa.

In addition, we stratified the analyses by country to study the socio-demographic correlates of sitting within the different countries. Because these single-country analyses had reduced statistical power, we only included the variables ‘Age’, ‘Gender’, ‘Age when stopped education’ and ‘Current occupation’ in these analyses. Because of the aforementioned co-linearity, we constructed the same two multivariate models. In order to compare the stratified analyses with similar overall results, we also conducted a general analysis including all country-specific variables and the variables ‘Age’, ‘Gender’, ‘Age when stopped education’ and ‘Current occupation’.

All data analyses were conducted in SPSS, version 22. We present ORs with 95% confidence intervals (95% CI). In addition, statistical significance of p<0.05 and p<0.001 is indicated.

## Results

Of the 27,919 respondents, 610 respondents (2.2 percent) were excluded because they were younger than 18 years, and 692 respondents (2.5 percent) were excluded because they had not answered the question on sitting time, leaving 26,617 respondents in the final analyses. People who answered ‘don’t know’ on the sitting question were on average 2.5 years older, and more often manual workers, house persons and retirees than people who did respond. There was no significant difference in gender or level of education. Mean age (SD) was 50.1 (17.5), 54.7 percent were female. All sample characteristics are shown in Table 1.

**Table 1:** Sample characteristics, median sitting time per day, and prevalence of sitting more than 7.5 hours per day, by country and socio-demographic characteristics.

	N (% total population)	Median (25^th^-75^th^ percentile) sitting minutes per day	N (within group %) sitting >7.5 hours per day
**Overall**	26617 (100%)	300 (180–420)	4924 (18.5%)
**Country (ref: all other countries)**			
Austria	1531 (5.8%)	300 (180–420)	280 (18.3%)
Belgium	1042 (3.9%)	300 (180–420)	186 (17.9%)
Bulgaria	947 (3.6%)	300 (240–420)	187 (19.7%)
Croatia	978 (3.7%)	300 (180–420)	222 (22.7%)
Czech Republic	985 (3.7%)	300 (180–480)	261 (26.5%)
Denmark	984 (3.7%)	360 (240–480)	312 (31.7%)
Estonia	983 (3.7%)	300 (180–420)	224 (22.8%)
Finland	942 (3.5%)	300 (240–420)	201 (21.3%)
France	991 (3.7%)	240 (180–420)	181 (18.3%)
Germany	948 (3.6%)	300 (240–420)	180 (19.0%)
Greece	973 (3.7%)	300 (180–420)	194 (19.9%)
Hungary	974 (3.7%)	240 (120–360)	115 (11.8%)
Ireland	953 (3.6%)	240 (180–360)	99 (10.4%)
Italy	955 (3.6%)	240 (180–360)	105 (11.0%)
Latvia	964 (3.6%)	300 (180–420)	168 (17.4%)
Lithuania	963 (3.6%)	300 (180–420)	161 (16.7%)
Luxembourg	484 (1.8%)	300 (180–420)	100 (20.7%)
Malta	488 (1.8%)	240 (120–360)	58 (11.9%)
Netherlands	991 (3.7%)	360 (240–480)	318 (32.1%)
Poland	879 (3.3%)	240 (180–360)	158 (18.0%)
Portugal	980 (3.7%)	180 (120–360)	98 (10.0%)
Republic of Cyprus	482 (1.8%)	300 (180–420)	92 (19.1%)
Romania	927 (3.5%)	240 (120–360)	133 (14.3%)
Slovakia	956 (3.6%)	300 (180–420)	190 (19.9%)
Slovenia	1094 (4.1%)	240 (120–300)	134 (12.2%)
Spain	982 (3.7%)	240 (180–360)	87 (8.9%)
Sweden	974 (3.7%)	300 (240–420)	236 (24.2%)
United Kingdom	1267 (4.8%)	300 (180–420)	244 (19.3%)
**Gender**			
Male	12062 (45.3%)	300 (180–420)	2357 (19.5%)
Female	14555 (54.7%)	300 (180–420)	2567 (17.6%)
**Age**			
18–24 years	2231 (8.4%)	300 (180–420)	459 (20.6%)
25–34 years	3852 (14.5%)	240 (180–420)	736 (19.1%)
35–44 years	4455 (16.7%)	240 (180–420)	843 (18.9%)
45–54 years	4786 (18.0%)	300 (180–420)	898 (18.8%)
55–64 years	4887 (18.4%)	300 (180–360)	807 (16.5%)
65+ years	6406 (24.1%)	300 (240–420)	1181 (18.4%)
**Marital status**			
Unmarried	4420 (16.8%)	300 (180–420)	927 (21.0%)
(Re-)Married/ with partner	17061 (65.0%)	300 (180–420)	2958 (17.3%)
Divorced or separated	2221 (8.5%)	300 (180–420)	429 (19.3%)
Widowed	2535 (9.7%)	300 (240–420)	520 (20.5%)
**Age when stopped education**			
Up to 15 years	4540 (17.4%)	240 (180–360)	629 (13.9%)
16–19 years	11794 (45.2%)	240 (180–360)	1830 (15.5%)
20+ years	8500 (32.6%)	300 (180–420)	2006 (23.6%)
Still studying	1270 (4.9%)	360 (300–480)	356 (28.0%)
**Current occupation**			
Self-employed	1971 (7.4%)	240 (180–360)	346 (17.6%)
*Farmer/fisherman*	*354 (1*.*3%)*	*180 (120–240)*	*9 (2*.*5%)*
*Professional*	*381 (1*.*4%)*	*360 (240–480)*	*112 (29*.*4%)*
*Owner of a shop/craftsman*	*716 (2*.*7%)*	*240 (180–360)*	*105 (14*.*7%)*
*Business proprietor*	*520 (2*.*0%)*	*300 (180–420)*	*120 (23*.*1%)*
Manager	2662 (10.0%)	360 (240–480)	806 (30.3%)
*Employed professional*	*688 (2*.*6%)*	*360 (240–480)*	*235 (34*.*2%)*
*General management*	*265 (1*.*0%)*	*360 (240–480)*	*96 (36*.*2%)*
*Middle management*	*1709 (6*.*4%)*	*360 (240–480)*	*475 (27*.*8%)*
Other white collar	3205 (12.0%)	360 (240–480)	1125 (35.1%)
*At desk*	*2376 (8*.*9%)*	*420 (300–540)*	*966 (40*.*7%)*
*Travelling*	*829 (3*.*1%)*	*240 (180–420)*	*159 (19*.*2%)*
Manual worker	5368 (20.2%)	240 (120–300)	481 (9.0%)
*Service job*	*1909 (7*.*2%)*	*240 (180–360)*	*193 (10*.*1%)*
*Supervisor*	*239 (0*.*9%)*	*240 (120–300)*	*22 (9*.*2%)*
*Skilled manual worker*	*2373 (8*.*9%)*	*240 (120–300)*	*207 (8*.*7%)*
*Unskilled manual worker*	*847 (3*.*2%)*	*240 (120–300)*	*59 (7*.*0%)*
House person	1929 (7.2%)	240 (120–300)	129 (6.7%)
Unemployed	2153 (8.1%)	240 (180–360)	243 (11.3%)
Retired	8059 (30.3%)	300 (180–420)	1438 (17.8%)
Student	1270 (4.8%)	360 (300–480)	356 (28.0%)
**Physical activity**			
Least active quartile	4863 (25.1%)	300 (180–420)	1150 (23.6%)
Second quartile	4830 (24.9%)	300 (180–420)	1023 (21.2%)
Third quartile	4834 (24.9%)	300 (180–360)	776 (16.1%)
Most active quartile	4852 (25.0%)	240 (180–300)	452 (9.3%)
**Type of community**			
Rural area or village	9124 (34.3%)	240 (180–360)	1357 (14.9%)
Small or medium sized town	9983 (37.5%)	300 (180–420)	1864 (18.7%)
Large town	7495 (28.2%)	300 (180–420)	1701 (22.7%)
**Children aged <15 years living in the household**			
None	19969 (75.0%)	300 (180–420)	3760 (18.8%)
One	3401 (12.8%)	240 (180–420)	628 (18.5%)
Two	2458 (9.2%)	240 (180–420)	443 (18.0%)
Three or more	789 (3.0%)	240 (120–360)	93 (11.8%)
**Television ownership**			
No	615 (2.3%)	300 (180–420)	130 (21.1%)
Yes	26002 (97.7%)	300 (180–420)	4794 (18.4%)
**Computer ownership**			
No	6819 (25.6%)	300 (180–360)	1079 (15.8%)
Yes	19798 (74.4%)	300 (180–420)	3845 (19.4%)
**Car ownership**			
No	7590 (28.5%)	300 (180–420)	1445 (19.0%)
Yes	19027 (71.5%)	300 (180–420)	3479 (18.3%)
**Internet use frequency**			
Everyday	15031 (56.5%)	300 (180–420)	3362 (22.4%)
Often/sometimes	4247 (16.0%)	240 (180–360)	457 (10.8%)
Never/no access	7339 (27.6%)	240 (180–360)	1105 (15.1%)
**Difficulties paying bills**			
Most of the time	3394 (13.0%)	240 (180–360)	544 (16.0%)
From time to time	7334 (28.0%)	240 (180–360)	1106 (15.1%)
Almost never/never	15455 (59.0%)	300 (180–420)	3182 (20.6%)
**Life satisfaction**			
Very satisfied	6282 (23.7%)	300 (180–420)	1266 (20.2%)
Fairly satisfied	14375 (54.3%)	300 (180–420)	2621 (18.2%)
Not very satisfied	4518 (17.1%)	240 (180–420)	742 (16.4%)
Not at all satisfied	1299 (4.9%)	300 (180–420)	263 (20.2%)

### Prevalence of sitting time

**Fig 2:**
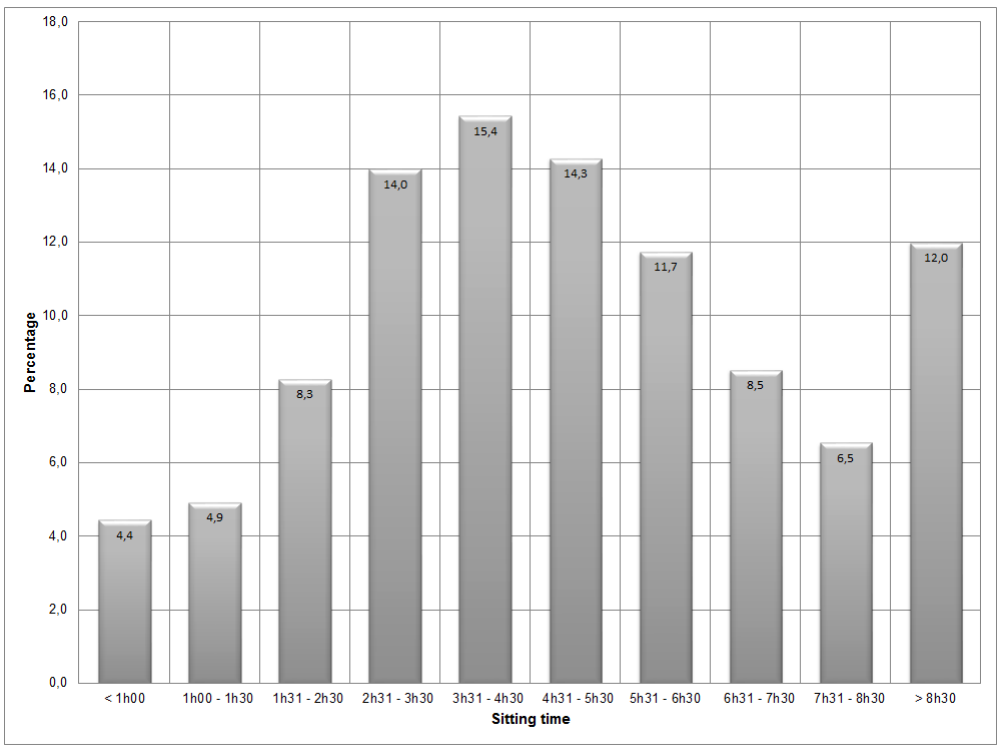
The distribution (%) of sitting time across the original response options, for all countries combined.

**Fig 3:**
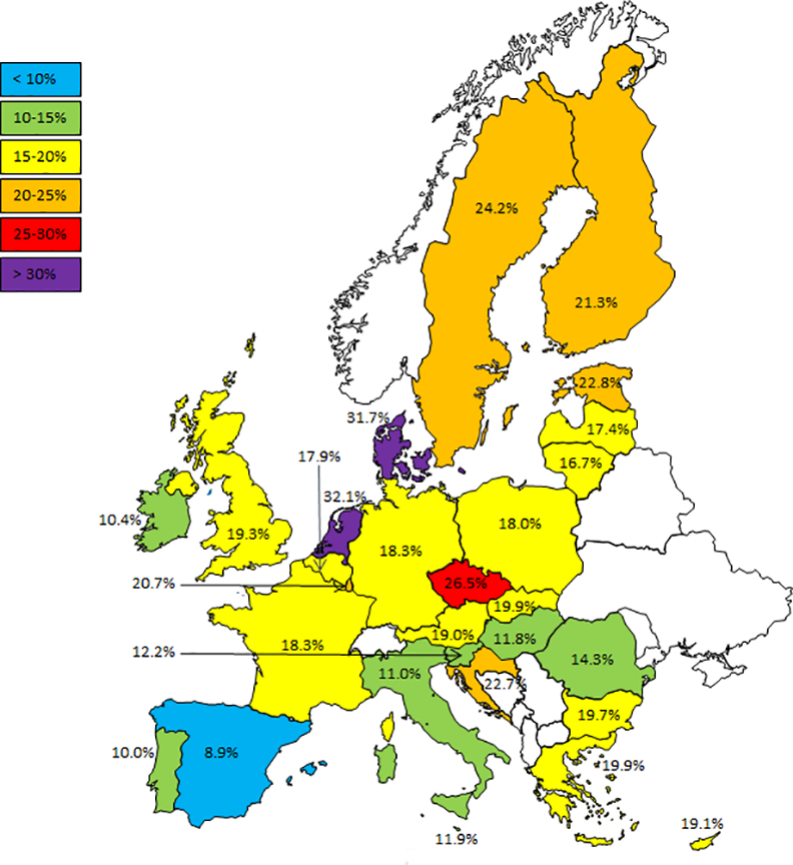
The distribution of the proportion of European adults reporting sitting more than 7.5 hours per day across the 28 European Union Member States. Designed by Showeet.com.

Fig 2 shows the distribution of sitting time across the original response options. The highest percentage (15.4 percent) can be found at 3.5–4.5 hours. The shape of the curve is roughly the same across all countries (data not shown). Overall, the respondents reported a median of 300 sitting minutes per day (interquartile range: 180–420), ranging from a median of 180 minutes in Portugal to 360 in Denmark and the Netherlands. In addition, 18.5 percent reported sitting 7.5 hours or more per day, ranging from 8.9 percent in Spain to 32.1 percent in the Netherlands (Table 1). The distribution of these proportions across Europe is shown in Fig 3. The geographical pattern on this map shows generally lower sitting percentages in the southern countries and higher percentages of people reporting more than 7.5 hours per day in the north of Europe.

### Correlates of sitting time

The results of the uni- and multivariate analyses are shown in Table 2. The highest ORs of sitting more than 7.5 hours per day were found regarding the occupation variable. White collar workers (OR: 5.00 (95% CI: 4.31–5.80)), students (3.84 (3.03–4.86)), managers (3.65 (3.13–4.25)), people who are self-employed (2.01 (1.67–2.42)), retirees (1.51 (1.25–1.82)) and people who are unemployed (1.31 (1.06–1.62)) all showed significantly higher ORs compared to manual workers, while house persons showed a lower OR of 0.72 (0.54–0.97). Students were also identified as a high-sitting group in the education variable, with an OR of 2.35 (1.81–3.04) of sitting more than 7.5 hours per day compared to people who were 15 years or younger when they stopped full-time education, as well as people who were 20 years or older when they stopped education (1.51 (1.28–1.79)). The multivariate models additionally show consistently significantly higher ORs of sitting more than 7.5 hours per day for (in descending order of effect size): people who use the internet every day (ref: never/no access) and people who are not at all satisfied with their life (ref: very satisfied. Consistently significantly lower ORs were found for (also in descending order of effect size): people in the most active quartile (ref: least active quartile), people in the third activity quartile, people with three or more children (ref: no children), people living in a rural area or village (ref: large town), people in the second activity quartile, people who experience difficulties paying their bills from time to time (ref: never), people who experience difficulties paying their bills most of the time, women (ref: men) and inhabitants of a small/medium sized town.

**Table 2:** Univariate and multivariate odds ratio (OR) of sitting more than 7.5 hours per day, by country and socio-demographic characteristics.

	Univariate OR (95% CI) of sitting >7.5 hours per day	Model 1: Multivariate^a^ OR (95% CI) of sitting >7.5 hours per day	Model 2: Multivariate^b^ OR (95% CI) of sitting >7.5 hours per day
**Country (ref: all other countries)**			
Netherlands	2.16 (1.88–2.47)**	2.01 (1.71–2.36)**	2.12 (1.80–2.50)**
Denmark	2.12 (1.84–2.43)**	1.55 (1.32–1.83)**	1.89 (1.60–2.23)**
Czech Republic	1.62 (1.40–1.87)**	1.86 (1.55–2.23)**	1.42 (1.18–1.71)**
Sweden	1.43 (1.23–1.66)**	1.13 (0.96–1.34)	1.08 (0.91–1.29)
Estonia	1.32 (1.13–1.53)**	1.20 (0.98–1.45)	1.16 (0.95–1.41)
Croatia	1.31 (1.12–1.52)*	1.47 (1.20–1.80)**	1.45 (1.18–1.77)**
Finland	1.20 (1.03–1.41)*	1.08 (0.90–1.30)	1.21 (1.01–1.46)*
Luxembourg	1.15 (0.92–1.44)	1.20 (0.93–1.56)	1.29 (0.99–1.68)
Greece	1.10 (0.94–1.29)	0.92 (0.71–1.20)	0.92 (0.71–1.20)
Slovakia	1.10 (0.93–1.29)	1.28 (1.05–1.57)*	1.18 (0.96–1.44)
Bulgaria	1.09 (0.92–1.28)	0.93 (0.67–1.28)	0.91 (0.65–1.26)
United Kingdom	1.05 (0.91–1.22)	0.80 (0.66–0.97)*	0.77 (0.64–0.94)*
Republic of Cyprus	1.04 (0.83–1.31)	0.77 (0.52–1.14)	0.75 (0.51–1.12)
Austria	1.03 (0.88–1.22)	1.07 (0.87–1.33)	0.87 (0.70–1.08)
Germany	0.99 (0.86–1.13)	1.03 (0.88–1.21)	1.02 (0.86–1.20)
France	0.98 (0.84–1.16)	0.93 (0.76–1.14)	1.04 (0.84–1.28)
Poland	0.96 (0.81–1.15)	0.95 (0.73–1.22)	0.98 (0.76–1.26)
Belgium	0.96 (0.81–1.12)	0.73 (0.60–0.90)*	0.73 (0.59–0.89)*
Latvia	0.93 (0.78–1.10)	0.93 (0.75–1.15)	0.95 (0.77–1.18)
Lithuania	0.88 (0.74–1.05)	0.81 (0.64–1.02)	0.87 (0.69–1.09)
Romania	0.73 (0.61–0.88)*	0.92 (0.69–1.22)	0.94 (0.70–1.26)
Slovenia	0.60 (0.50–0.73)**	0.57 (0.46–0.72)**	0.58 (0.46–0.73)**
Malta	0.59 (0.45–0.78)**	0.87 (0.57–1.33)	0.86 (0.56–1.34)
Hungary	0.58 (0.48–0.71)**	0.54 (0.41–0.72)**	0.51 (0.38–0.67)**
Italy	0.53 (0.44–0.66)**	0.68 (0.51–0.90)*	0.57 (0.43–0.76)**
Ireland	0.50 (0.41–0.62)**	0.49 (0.38–0.64)**	0.54 (0.41–0.71)**
Portugal	0.48 (0.39–0.59)**	0.41 (0.28–0.62)**	0.39 (0.26–0.58)**
Spain	0.42 (0.34–0.52)**	0.46 (0.34–0.62)**	0.51 (0.37–0.69)**
**Gender**			
Male (ref)	1.00	1.00	1.00
Female	0.88 (0.83–0.94)**	0.81 (0.74–0.87)**	0.81 (0.74–0.88)**
**Age**			
*Overall*	*0*.*97 (0*.*95–0*.99)*	*0*.*94 (0*.*91–0*.98)*	*0*.*92 (0*.*89–0*.95)**
18–24 years	1.10 (0.96–1.25)	0.75 (0.62–0.92)*	0.87 (0.71–1.06)
25–34 years (ref)	1.00	1.00	1.00
35–44 years	0.99 (0.89–1.10)	1.08 (0.94–1.24)	1.02 (0.89–1.18)
45–54 years	0.98 (0.88–1.09)	1.08 (0.94–1.24)	1.04 (0.90–1.20)
55–64 years	0.84 (0.75–0.94)*	0.80 (0.68–0.93)*	0.90 (0.76–1.06)
65+ years	0.96 (0.86–1.06)	0.66 (0.55–0.78)**	0.87 (0.70–1.08)
**Marital status**			
Unmarried (ref)	1.00	1.00	1.00
(Re-)Married/ with partner	0.79 (0.73–0.86)**	0.97 (0.86–1.10)	0.97 (0.86–1.10)
Divorced or separated	0.90 (0.79–1.03)	1.02 (0.85–1.21)	1.01 (0.84–1.21)
Widowed	0.97 (0.86–1.10)	1.21 (0.98–1.49)	1.18 (0.95–1.46)
**Age when stopped education**			
*Overall*	*1*.*43 (1*.*38–1*.49)**	*1*.*32 (1*.*24–1*.40)**	
Up to 15 years (ref)	1.00	1.00	
16–19 years	1.14 (1.04–1.26)*	1.04 (0.89–1.23)	
20+ years	1.92 (1.74–2.12)**	1.51 (1.28–1.79)**	
Still studying	2.42 (2.09–2.81)**	2.35 (1.81–3.04)**	
**Current occupation**			
Self-employed	2.16 (1.86–2.51)**		2.01 (1.67–2.42)**
Manager	4.41 (3.89–5.00)**		3.65 (3.13–4.25)**
Other white collar	5.50 (4.88–6.19)**		5.00 (4.31–5.80)**
Manual worker (ref)	1.00		1.00
House person	0.73 (0.60–0.89)*		0.72 (0.54–0.97)*
Unemployed	1.29 (1.10–1.52)*		1.31 (1.06–1.62)*
Retired	2.21 (1.98–2.46)**		1.51 (1.25–1.82)**
Student	3.96 (3.39–4.62)**		3.84 (3.03–4.86)**
**Physical activity**			
*Overall*	*0*.*71 (0*.*69–0*.74)**	*0*.*66 (0*.*64–0*.69)**	*0*.*66 (0*.*63–0*.68)**
Least active quartile	1.00	1.00	1.00
Second quartile	0.87 (0.79–0.96)*	0.73 (0.66–0.81)**	0.73 (0.66–0.81)**
Third quartile	0.62 (0.56–0.68)**	0.50 (0.44–0.55)**	0.50 (0.45–0.56)**
Most active quartile	0.33 (0.30–0.37)**	0.27 (0.24–0.31)**	0.29 (0.26–0.33)**
**Type of community**			
*Overall*	*1*.*30 (1*.*25–1*.35)**	*1*.*24 (1*.*18–1*.31)**	*1*.*26 (1*.*19–1*.32)**
Rural area or village	0.60 (0.55–0.64)**	0.66 (0.60–0.74)**	0.71 (0.63–0.79)**
Small or medium sized town	0.78 (0.73–0.84)**	0.80 (0.73–0.89)**	0.85 (0.77–0.94)*
Large town (ref)	1.00	1.00	1.00
**Children aged <15 years living in the household**			
*Overall*	*0*.*92 (0*.*89–0*.96)**	*0*.*97 (0*.*92–1*.*02)*	*0*.*90 (0*.*85–0*.95)**
None (ref)	1.00	1.00	1.00
One	0.98 (0.89–1.07)	0.98 (0.87–1.12)	1.03 (0.90–1.17)
Two	0.95 (0.85–1.06)	0.94 (0.81–1.09)	0.96 (0.82–1.12)
Three or more	0.58 (0.46–0.72)**	0.66 (0.51–0.86)*	0.70 (0.53–0.92)*
**Television ownership**			
No (ref)	1.00	1.00	1.00
Yes	0.84 (0.69–1.03)	0.89 (0.70–1.15)	0.88 (0.69–1.13)
**Computer ownership**			
No (ref)	1.00	1.00	1.00
Yes	1.28 (1.19–1.38)**	0.91 (0.76–1.09)	0.93 (0.77–1.13)
**Car ownership**			
No (ref)	1.00	1.00	1.00
Yes	0.95 (0.89–1.02)	0.95 (0.85–1.06)	0.88 (0.78–0.98)*
**Internet use frequency**			
*Overall*	*0*.*75 (0*.*72–0*.78)**	*0*.*62 (0*.*57–0*.68)**	*0*.*63 (0*.*57–0*.68)**
Everyday	1.63 (1.51–1.75)**	1.78 (1.46–2.18)**	1.53 (1.24–1.88)**
Often/sometimes	0.68 (0.61–0.76)**	0.81 (0.65–1.00)	0.84 (0.67–1.04)
Never/no access (ref)	1.00	1.00	1.00
**Difficulties paying bills**			
*Overall*	*1*.*24 (1*.*19–1*.30)**	*1*.*22 (1*.*13–1*.32)**	*1*.*24 (1*.*15–1*.33)**
Most of the time	0.74 (0.67–0.81)**	0.75 (0.64–0.89)*	0.83 (0.70–0.98)*
From time to time	0.69 (0.64–0.74)**	0.73 (0.66–0.81)**	0.75 (0.68–0.84)**
Almost never/never (ref)	1.00	1.00	1.00
**Life satisfaction**			
*Overall*	*0*.*94 (0*.*90–0*.98)*	*1*.*08 (1*.*01–1*.15)*	*1*.*06 (0*.*99–1*.*13)*
Very satisfied (ref)	1.00	1.00	1.00
Fairly satisfied	0.88 (0.82–0.95)*	1.05 (0.95–1.16)	1.07 (0.97–1.19)
Not very satisfied	0.78 (0.70–0.86)**	1.08 (0.93–1.27)	1.19 (1.01–1.40)*
Not at all satisfied	1.01 (0.87–1.17)	1.37 (1.04–1.81)*	1.55 (1.17–2.05)*

The countries are ordered based on the univariate OR. Because of co-linearity between the Education and Occupation variables we constructed two multivariate models: one including Education excluding Occupation (1) and vice versa (2).

* p<0.05

** p<0.001

a. Adjusted for country, gender, age, marital status, age when stopped education, physical activity, type of community, children aged <15 years living in the household, television ownership, computer ownership, car ownership, internet use frequency, difficulties paying bills and life satisfaction.

b. Adjusted for country, gender, age, marital status, current occupation, physical activity, type of community, children aged <15 years living in the household, television ownership, computer ownership, car ownership, internet use frequency, difficulties paying bills and life satisfaction.

### Single-country analyses

The analyses stratified by country are shown in S1 Table and Tables A-BB in S1 File. In general, these analyses show that the above-mentioned associations are fairly consistent across the EU countries. This is most visible for the occupation variable. White collar workers show substantial as well as statistical significant higher ORs in all 28 countries and managers, students, self-employed, retired and unemployed show higher ORs in the majority of countries, although not all statistically significant. House persons were shown to have a higher OR in half of the countries while the other half showed lower ORs, with no obvious geographic pattern.

In comparison to people who were up to 15 years when they stopped full-time education, people who were 20 years or older when they stopped full-time education and people whose current occupation was “still studying” showed higher ORs in almost all countries, with approximately one-third of the countries showing a significant association. Most countries indicated a lower OR for people who were between 16 and 19 years old when they stopped education; the countries indicating a higher OR for this group were mainly countries in the south-east of Europe.

Approximately two thirds of the countries showed a lower OR for people aged 18–24 years as compared to people who were 25–34 years old. The countries indicating a higher OR for this group were mainly situated in the south-eastern part of Europe. In addition, two-thirds of the countries also showed lower OR for people aged 55–64 years, with no obvious geographic pattern. The countries showed a 50/50 distribution for the other age groups. The above-mentioned lower ORs for women were also visible in approximately two-thirds of the countries. The countries indicating the opposite were mainly southern European countries.

## Discussion

In this study we explored the prevalence and correlates of self-reported sitting time in adults across and within the 28 EU Member States. The data show striking differences in the prevalence of sitting across countries, with extremes ranging from 9 percent of the Spanish respondents reporting sitting more than 7.5 hours per day, to 32 percent of the Dutch. In general, the northern European countries reported more sitting, while the southern countries reported sitting less. We identified several socio-demographic characteristics that were associated with sitting time. The strongest associations were found for current occupation and level of education, with white collar occupations being five times more likely to report sitting more than 7.5 hours per day compared to manual workers. When stratifying the analyses by country, the associations between occupation, and to a lesser extent education, and self-reported sitting time proved reasonably consistent within most EU countries.

### Prevalence of sitting time

Respondents to the 2013 Eurobarometer indicated a median of 300 sitting minutes per day, which is the same as the 2005 Eurobarometer sample[20] and a worldwide 20-country comparison conducted by Bauman and colleagues.[23] However, when Milton and colleagues[18] recently studied the trends in self-reported sitting time based on the 2002, 2005 and 2013 Eurobarometer data, they concluded that the prevalence of sitting more than 7.5 hours per day had decreased between 2002 and 2013. This unexpected result highlights the importance of regular monitoring of SB.

When comparing the self-reported results of this Eurobarometer with objectively measured population levels of sitting time, the Eurobarometer seems to underestimate actual sitting time, even when taking possible temporal changes into account. For example, the United Kingdom respondents of the Eurobarometer reported a median of 300 sitting minutes, while the accelerometer data of the 2008 Health Survey of England showed an average of 584 daily sitting minutes for women and 595 for men [24]. A similar, but smaller, difference can be seen in Sweden, where accelerometer data showed a mean of 459 sitting minutes per day, while Swedish Eurobarometer respondents reported a median of 300 minutes in 2001.[25] The difference is even more remarkable in Portugal, with 180 median minutes based on the Eurobarometer, against a range of 529 to 612 average sitting minutes based on accelerometer data from 2006–2008.[26] Moreover, a Norwegian validation study in a national sample concluded that people on average report two hours less sitting time with the IPAQ-short compared to the ActiGraph accelerometer.[27] These results suggest that not only the absolute numbers, but also the relative order of countries might be dependent on the instrument used, and that underreporting may be more prevalent in some countries than in others. This observation shows that more research in this field is warranted.

We observed a north-south division across Europe, with southern countries reporting less sitting and northern countries sitting more, even when adjusted for socio-demographic variables. Notable exceptions to this observation are Ireland (with 10.4 percent of the respondents reporting sitting more than 7.5 hours per day), Croatia and the Czech Republic (22.7 and 26.5 percent, respectively). The previous Eurobarometer studies reported a similar geographical pattern.[19,20] These findings could reflect real differences between countries, possibly caused by differences in wealth, culture or climate. In this light, it should be noted that all three Eurobarometer surveys were conducted from October to December, a time in which people in northern Europe might be more inclined to stay in, use motorised transportation and replace PA with SB. Alternatively, the observed differences could also be caused by differences in recall- and/or social desirability bias between countries. For example, people living in countries with more public health campaigns against sitting might be more inclined to underreport their sitting time. As there is no literature to support these hypotheses, more research is needed to investigate the differences in sitting time across Europe.

### Correlates of sitting time

Our multivariate analyses showed that women have a lower OR of sitting more than 7.5 hours per day than men. These findings are in line with the previous Eurobarometer studies.[19,20] However, the International Prevalence Study (IPS)[23] found no gender differences in the adjusted models and a review by Rhodes et al.[13] reported no association for gender and general sitting behaviour in the majority of studies, although men were shown to sit more in two studies.

With regard to age, one of our multivariate analyses showed that people aged 55–64 years and people aged 65+ years have a lower OR of reporting sitting more than 7.5 hours per day than people aged 25–34 years. This is in line with previous Eurobarometer studies[19,20] and IPS[23], who all reported that younger people sat more. However, the same model also shows that people aged 18–24 have a lower OR than people aged 25–34. Since our univariate analysis shows a (non-significant) higher OR for people aged 18–24 years, a possible explanation for our multivariate results might be that a large proportion of this group are studying/students, which could be more strongly related to sitting than age itself. Notably, Rhodes et al.[13] reported ‘extremely mixed’ results for age and sitting time, suggesting no clear cut association.

One of the more novel socio-demographic variables in our analysis was marital status. The other studies[19,20,23] did not include this variable in their analysis, and Rhodes et al.[13] concluded more evidence is needed. However, we did not find any associations in our multivariate models. People who are married/with a partner showed a significant lower OR in the univariate model, but not in the multivariate models.

People with higher education levels are consistently identified as more likely to be high-sitters.[19,20,23] This is possibly related to a higher prevalence of sedentary occupations in higher educated people. Occupation was found to be the strongest correlate of self-reported sitting time. All occupations have higher ORs than manual workers, except for house persons. The review of Rhodes et al.[13] confirms that unemployed and retired people usually sit more, but did not report on the other groups. Our findings suggests that sedentary occupations are important predictors of high overall sitting time. This highlights the importance to target SB at the workplace. A recent systematic literature review and meta-analysis by Neuhaus et al. concluded that so-called activity permissive workstations (e.g. standing desks, treadmill desks, etc) can be effective in decreasing occupational SB, without affecting work performance.[28]

Our findings regarding physical activity, type of community and number of children are in line with previous research.[13,19,20,23] With regard to the children, Rhodes et al.[13] concluded that “the presence of children is associated with less SB.” However, our results seem to suggest this association is only present in families with three or more children, which might suggest having more children is associated with more daily responsibilities and less opportunities to sit down.

No associations were found between television and computer ownership and sitting time, and car owners show a significantly lower OR of sitting more than 7.5 hours per day compared to people without a car in one of the models. These findings are counter-intuitive, since time driving in a car, watching television and using a computer is usually spent sitting down. However, this might have been a measurement issue, as respondents were asked whether or not they owned a television, computer and car, and not how much time they spent using it. This hypotheses is strengthened by the observation that habitual internet users did show higher ORs, when compared to people who never use the internet or have no access.

We found that people who reported having difficulties paying bills “from time to time” have lower ORs than people who “almost never/never” experience these difficulties. In addition, people who experience these difficulties “most of the time” show even lower ORs. Since this variable is related to the socio-economic status of the respondents, it is not surprising these results are in line with the education and occupation variables.

Finally, people with lower life satisfaction have higher ORs of sitting more than 7.5 hours per day, showing a dose-response relationship. This association could go both ways; people with high levels of sitting might be less satisfied with their life; and people with low life satisfaction might be more prone to sitting. This association could also be explained by the health status of the respondent, e.g. people with illness/depression are often more sedentary and less satisfied with their life. This is supported by a review by Teychenne et al.[29], who concluded that the available evidence suggests that SB is associated with a higher risk of depression.

### Country specific results

The analyses stratified by country showed that the associations between type of occupation, and to a lesser extent, educational level, and sitting time are similar across European countries, while this was less clear for the age and gender variables. This finding suggests the need to target individuals with sedentary occupations in interventions aiming to reduce sitting time throughout Europe.

In addition, for some socio-demographic groups there seemed to be different associations within south-eastern or eastern European countries in the single-country analyses. Although this is only a first impression, this might indicate that different groups engage in high sitting time in these countries, which could have implications for policy and need to be investigated further.

### Strengths and limitations

The response rate and representativeness of the Eurobarometer are our first point of concern. The average response rate was 46 percent, ranging from 11 to 80 percent. Several procedures were used to maximize response rates, such as using experienced interviewers, reviewing the training materials and methods, and multiple call-backs on different days of the week and times of the day. The origin of the variation in response rates across countries is unclear. This response rate, together with the high mean age (52 years) of the sample, provide an indication that the study sample is not representative for the adult population at large. This might be especially true for the countries with the lowest response rates.

The main limitation of this Eurobarometer survey, however, lies with the assessment of sitting time and PA. The survey included an adapted version of the IPAQ-short,[21] a questionnaire that is widely used and has reasonable validity and reliability.[22,30] The developers of the Eurobarometer survey adapted the IPAQ-short so that respondents were asked to choose between several categorical response options, with a range of 60 (sitting time) or 30 (PA) minutes, as opposed to the original continuous reporting in hours and minutes. Unfortunately, the response categories for PA were adapted from the original IPAQ in such a way that it was impossible to accurately distinguish people that met PA recommendations from people that did not. In addition, it prevented us from accurately estimating the time respondents spend on PA. For this reason, we used the midpoint of each category as a rough estimate of the time spend in PA, and constructed quartiles to compare these relative rather than absolute values.

Even though the IPAQ-short sitting question response categories were acceptable, the self-report nature of the sitting time assessment remains a limitation of this study. Self-reported sitting time is subject to recall and social desirability bias, which most likely results in underestimations of sitting time.[31] Furthermore, possible cultural and language differences in these biases and in the interpretation of the questionnaire might complicate comparison between countries. In addition, the question only asks about a ‘usual day’, which means it was not possible to assess differences in sitting time between week- and weekend days.

Even though these limitations should be taken into account in the interpretation of the findings, the Eurobarometer is currently the only source of comparable sitting time data across all 28 EU Member States, providing the opportunity to study and compare current levels of SB and its correlates.

### Conclusions

There is substantial variation in the prevalence of sitting among European adults across countries and socio-demographic groups. Across Europe, a geographical pattern can be observed, with northern countries reporting more sitting and southern countries reporting less. Non-blue collar occupations and higher educational levels were found to be the strongest correlates of higher levels of sitting time. In addition, women, physically active people, people living in a rural area or in a small/medium sized town, people with three or more children, infrequent internet users, people with difficulties paying their bills, and people who are satisfied with their life report lower sitting levels. The observed associations between occupation, and to a lesser extent education, and sitting were reasonably consistent across Europe.

The Eurobarometer is currently the only source of comparable sitting data across Europe, but its measures of sitting time have been irregular across time and inconsistent between consecutive surveys. In addition, the self-report nature of the measurement of sitting used in the Eurobarometer surveys may introduce bias. To enable monitoring of population levels, identification of populations at risk and targeted actions for these populations, regular surveillance of population levels of SB is needed, using identical methodologies over time, and preferably including objective measures of SB. In addition, more research should be focussed on the correlates and determinants of SB since they are mainly unknown. The results of this study provide entry points for targeted interventions aimed at high-sitting populations, such as people with sedentary occupations.

## Supporting Information

S1 File Sample characteristics and prevalence, univariate and multivariate odds ratio (OR) of sitting more than 7.5 hours per day, by gender, age, education and occupation, separately for the 28 European Union Member States (Tables A-BB). Because of co-linearity between the Education and Occupation variables we constructed two multivariate models: one including Education excluding Occupation (Model 1) and vice versa (Model 2).
(DOCX)

S1 Table Sample characteristics and prevalence, univariate and multivariate odds ratio (OR) of sitting more than 7.5 hours per day, by country and socio-demographic characteristics. The countries are ordered based on the univariate OR. Because of co-linearity between the Education and Occupation variables we constructed two multivariate models: one including Education excluding Occupation (Model 1) and vice versa (Model 2).
(DOCX)

## References

[1] Sedentary Behaviour Research Network. Letter to the Editor: Standardized use of the terms “sedentary” and “sedentary behaviours.” Appl Physiol Nutr Metab. 2012;37: 540–542. 10.1139/H2012-024 22540258

[2] World Health Organization. Global recommendations on physical activity for health [Internet]. World Health Organization 2010 10.1080/11026480410034349 26180873

[3] Wilmot EG, Edwardson CL, Achana FA, Davies MJ, Gorely T, Gray LJ, et al Sedentary time in adults and the association with diabetes, cardiovascular disease and death: Systematic review and meta-analysis. Diabetologia. 2012;55: 2895–2905. 10.1007/s00125-012-2677-z 22890825

[4] Proper KI, Singh AS, Van Mechelen W, Chinapaw MJM. Sedentary behaviors and health outcomes among adults: A systematic review of prospective studies. Am J Prev Med. Elsevier Inc.; 2011;40: 174–182. 10.1016/j.amepre.2010.10.015 21238866

[5] Grøntved A, Hu FB. Television viewing and risk of type 2 diabetes, cardiovascular disease, and all-cause mortality: a meta-analysis. Jama. 2011;305: 2448–55. 10.1001/jama.2011.812 21673296 PMC4324728

[6] Van Uffelen JGZ, Wong J, Chau JY, Van Der Ploeg HP, Riphagen I, Gilson ND, et al Occupational sitting and health risks: A systematic review. Am J Prev Med. 2010;39: 379–388. 10.1016/j.amepre.2010.05.024 20837291

[7] Ford ES, Caspersen CJ. Sedentary behaviour and cardiovascular disease: A review of prospective studies. Int J Epidemiol. 2012;41: 1338–1353. 10.1093/ije/dys078 22634869 PMC4582407

[8] Thorp AA, Owen N, Neuhaus M, Dunstan DW. Sedentary behaviors and subsequent health outcomes in adults: A systematic review of longitudinal studies, 1996–2011. Am J Prev Med. Elsevier Inc.; 2011;41: 207–215. 10.1016/j.amepre.2011.05.004 21767729

[9] Chau JY, Grunseit AC, Chey T, Stamatakis E, Brown WJ, Matthews CE, et al Daily sitting time and all-cause mortality: A meta-analysis. PLoS One. 2013;8: e80000 10.1371/journal.pone.0080000 24236168 PMC3827429

[10] Bauman AE, Chau JY, Ding D, Bennie J. Too Much Sitting and Cardio-Metabolic Risk: An Update of Epidemiological Evidence. Curr Cardiovasc Risk Rep. 2013;7: 293–298. 10.1007/s12170-013-0316-y

[11] De Rezende LFM, Rey-López JP, Matsudo VKR, do Carmo Luiz O. Sedentary behavior and health outcomes among older adults: a systematic review. BMC Public Health. 2014;14: 333 10.1186/1471-2458-14-333 24712381 PMC4021060

[12] Hallal PC, Andersen LB, Bull FC, Guthold R, Haskell W, Ekelund U, et al Physical Activity 1 Global physical activity levels: surveillance progress, pitfalls,. Lancet. 2012;380: 247–257. 10.1016/S0140-6736(12)60646-1 22818937

[13] Rhodes RE, Mark RS, Temmel CP. Adult sedentary behavior: A systematic review. Am J Prev Med. Elsevier Inc.; 2012;42: e3–e28. 10.1016/j.amepre.2011.10.020 22341176

[14] European Commission. Eurobarometer surveys [Internet]. Available: http://ec.europa.eu/public_opinion/index_en.htm

[15] European Commission. Special Eurobarometer 183–6: Physical Activity (October-December 2002). 2003.

[16] European Commission. Special Eurobarometer 246: Health and food (November-December 2005). 2006. 10.1016/S0015-6264(73)80341-4

[17] European Commission. Special Eurobarometer 412: Sport and physical activity (November-December 2013). 2014.

[18] Milton K, Gale J, Stamatakis E, Bauman A. Trends in prolonged sitting time among European adults: 27 country analysis. Prev Med (Baltim). 2015; 10.1016/j.ypmed.2015.04.016 25937588

[19] Sjöström M, Oja P, Hagströmer M, Smith BJ, Bauman A. Health-enhancing physical activity across European Union countries: The Eurobarometer study. J Public Health (Bangkok). 2006;14: 291–300. 10.1007/s10389-006-0031-y

[20] Bennie JA, Chau JY, van der Ploeg HP, Stamatakis E, Do A, Bauman A. The prevalence and correlates of sitting in European adults—a comparison of 32 Eurobarometer-participating countries. Int J Behav Nutr Phys Act. International Journal of Behavioral Nutrition and Physical Activity; 2013;10: 107 10.1186/1479-5868-10-107 24020702 PMC3847463

[21] The IPAQ group. International Physical Activity Questionnaire (IPAQ) [Internet]. Available: https://sites.google.com/site/theipaq/

[22] Rosenberg DE, Bull FC, Marshall AL, Sallis JF, Bauman AE. Assessment of sedentary behavior with the International Physical Activity Questionnaire. J Phys Act Health. 2008;5 Suppl 1: S30–S44. PAA0006 18364524 10.1123/jpah.5.s1.s30

[23] Bauman A, Ainsworth BE, Sallis JF, Hagströmer M, Craig CL, Bull FC, et al The descriptive epidemiology of sitting: A 20-country comparison using the international physical activity questionnaire (IPAQ). Am J Prev Med. 2011;41: 228–235. 10.1016/j.amepre.2011.05.003 21767731

[24] Craig R, Mindell J, Hirani V. Health Survey for England 2008. Volume 1: Physical activity and fitness. 2009.

[25] Hagströmer M, Oja P, Sjöström M. Physical activity and inactivity in an adult population assessed by accelerometry. Med Sci Sports Exerc. 2007;39: 1502–1508. 10.1249/mss.0b013e3180a76de5 17805081

[26] Baptista F, Santos DA, Silva AM, Mota J, Santos R, Vale S, et al Prevalence of the portuguese population attaining sufficient physical activity. Med Sci Sports Exerc. 2012;44: 466–473. 10.1249/MSS.0b013e318230e441 21844823

[27] Hansen BH, Kolle E, Dyrstad SM, Holme I, Anderssen SA. Accelerometer-determined physical activity in adults and older people. Med Sci Sports Exerc. 2012;44: 266–272. 10.1249/mss.0b013e31822cb354 21796052

[28] Neuhaus M, Eakin EG, Straker L, Owen N, Dunstan DW, Reid N, et al Reducing occupational sedentary time: a systematic review and meta-analysis of evidence on activity-permissive workstations. Obes Rev. 2014; 15: 822–838. 10.1111/obr.12201 25040784

[29] Teychenne M, Ball K, Salmon J. Sedentary behavior and depression among adults: A review. Int J Behav Med. 2010;17: 246–254. 10.1007/s12529-010-9075-z 20174982

[30] Craig CL, Marshall AL, Sjöström M, Bauman AE, Booth ML, Ainsworth BE, et al International physical activity questionnaire: 12-Country reliability and validity. Med Sci Sports Exerc. 2003;35: 1381–1395. 10.1249/01.MSS.0000078924.61453.FB 12900694

[31] Healy GN, Clark BK, Winkler EAH, Gardiner PA, Brown WJ, Matthews CE. Measurement of adults’ sedentary time in population-based studies. Am J Prev Med. Elsevier Inc.; 2011;41: 216–227. 10.1016/j.amepre.2011.05.005 21767730 PMC3179387

