# Supplementary material for: European Sitting Championship: Prevalence and Correlates of Self-Reported Sitting Time in the 28 European Union Member States
Source: PLoS One. 2016 Mar 2;11(3):e0149320. doi: 10.1371/journal.pone.0149320 (PMC4774909; doi:10.1371/journal.pone.0149320)
Supplement: S1 File — Because of co-linearity between the Education and Occupation variables we constructed two multivariate models: one including Education excluding Occupation (Model 1) and vice versa (Model 2). (DOCX) [file pone.0149320.s001.docx]

S1 File, Table A. Sample characteristics and prevalence, univariate and multivariate odds ratio (OR) of sitting more than 7.5 hours per day, by gender, age, education and occupation, in Austria. Because of co-linearity between the Education and Occupation variables we constructed two multivariate models: one including Education excluding Occupation (Model 1) and vice versa (Model 2).

| **Austria** | **N (% total population)** | **Median (25^th^-75^th^ percentile) sitting minutes per day** | **N (within group %) sitting >7.5 hours per day** | **Univariate OR (95% CI) of sitting >7.5 hours per day** | **Model 1: Multivariate^b^ OR (95% CI) of sitting >7.5 hours per day** | **Model 2: Multivariate^a^ OR (95% CI) of sitting >7.5 hours per day** |
| --- | --- | --- | --- | --- | --- | --- |
| **Overall** | 948(100%) | 300(240-420) | 180(19%) | - | - | - |
| **Gender**  Male (ref)  Female | 448(47.3%)  500(52.7%) | 300(240-420)  360(240-420) | 78(17.4%)  102(20.4%) | 1.00  1.22(0.88-1.69) | 1.00  1.22(0.87-1.70) | 1.00  1.06(0.75-1.51) |
| **Age**  18-24 years  25-34 years (ref)  35-44 years  45-54 years  55-64 years  65+ years | 80(8.4%)  153(16.1%)  174(18.4%)  207(21.8%)  154(16.2%)  180(19%) | 360(240-480)  300(240-420)  300(240-420)  300(240-420)  300(240-420)  360(240-420) | 20(25%)  32(20.9%)  33(19%)  39(18.8%)  20(13%)  36(20%) | 1.26(0.67-2.39)  1.00  0.88(0.51-1.52)  0.88(0.52-1.48)  0.56(0.31-1.04)  0.95(0.55-1.61) | 1.95(0.95-4.01)  1.00  0.86(0.50-1.49)  0.88(0.52-1.50)  0.57(0.30-1.07)  1.10(0.63-1.91) | 1.73(0.84-3.57)  1.00  0.81(0.47-1.43)  0.92(0.54-1.58)  0.74(0.37-1.45)  1.75(0.7-4.39) |
| **Age stopped education**  Up to 15 years (ref)  16-19 years  20+ years  Still studying | 247(26.8%)  487(52.8%)  146(15.8%)  42(4.6%) | 300(240-360)  360(240-420)  360(240-420)  300(240-420) | 29(11.7%)  109(22.4%)  33(22.6%)  6(14.3%) | 1.00  2.17(1.39-3.37)*  2.20(1.27-3.80)*  1.25(0.49-3.23) | 1.00  2.26(1.44-3.55)**  2.38(1.35-4.20)*  0.72(0.25-2.11) |  |
| **Occupation**  Self-employed  Managers  Other white collars  Manual workers (ref)  House persons  Unemployed  Retired  Students | 56(5.9%)  166(17.5%)  130(13.7%)  239(25.2%)  42(4.4%)  26(2.7%)  247(26.1%)  42(4.4%) | 300(240-360)  360(255-480)  360(300-480)  240(180-360)  300(180-420)  360(285-420)  300(240-420)  300(240-420) | 10(17.9%)  41(24.7%)  43(33.1%)  29(12.1%)  8(19%)  2(7.7%)  41(16.6%)  6(14.3%) | 1.57(0.72-3.46)  2.38(1.41-4.01)*  3.58(2.10-6.10)**  1.00  1.70(0.72-4.04)  0.60(0.14-2.69)  1.44(0.86-2.41)  1.21(0.47-3.11) |  | 1.75(0.79-3.87)  2.47(1.46-4.19)*  3.56(2.05-6.19)**  1.00  1.72(0.7-4.2)  0.61(0.14-2.77)  0.97(0.42-2.26)  0.75(0.26-2.17) |

*p<0.05 **p<0.001

S1 File, Table B. Sample characteristics and prevalence, univariate and multivariate odds ratio (OR) of sitting more than 7.5 hours per day, by gender, age, education and occupation, in Belgium. Because of co-linearity between the Education and Occupation variables we constructed two multivariate models: one including Education excluding Occupation (Model 1) and vice versa (Model 2).

| **Belgium** | **N (% total population)** | **Median (25^th^-75^th^ percentile) sitting minutes per day** | **N (within group %) sitting >7.5 hours per day** | **Univariate OR (95% CI) of sitting >7.5 hours per day** | **Model 1: Multivariate^b^ OR (95% CI) of sitting >7.5 hours per day** | **Model 2: Multivariate^a^ OR (95% CI) of sitting >7.5 hours per day** |
| --- | --- | --- | --- | --- | --- | --- |
| **Overall** | 1042(100%) | 300(180-420) | 186(17.9%) | - | - | - |
| **Gender**  Male (ref)  Female | 506(48.6%)  536(51.4%) | 300(180-420)  300(180-420) | 99(19.6%)  87(16.2%) | 1.00  0.80(0.58-1.10) | 1.00  0.78(0.56-1.09) | 1.00  0.81(0.58-1.15) |
| **Age**  18-24 years  25-34 years (ref)  35-44 years  45-54 years  55-64 years  65+ years | 83(8%)  151(14.5%)  166(15.9%)  198(19%)  197(18.9%)  247(23.7%) | 360(240-480)  300(180-420)  240(180-360)  300(180-360)  300(180-360)  300(240-420) | 30(36.1%)  31(20.5%)  25(15.1%)  32(16.2%)  26(13.2%)  42(17%) | 2.19(1.21-3.98)*  1.00  0.69(0.38-1.23)  0.75(0.43-1.29)  0.59(0.33-1.04)  0.79(0.47-1.33) | 0.87(0.35-2.17)  1.00  0.70(0.39-1.27)  0.83(0.48-1.45)  0.65(0.36-1.18)  0.94(0.54-1.65) | 0.83(0.32-2.16)  1.00  0.61(0.33-1.13)  0.68(0.38-1.22)  0.77(0.4-1.47)  1.55(0.68-3.54) |
| **Age stopped education**  Up to 15 years (ref)  16-19 years  20+ years  Still studying | 124(12.1%)  418(40.7%)  438(42.6%)  48(4.7%) | 300(180-420)  300(180-360)  300(180-420)  480(300-540) | 20(16.1%)  47(11.2%)  92(21%)  25(52.1%) | 1.00  0.66(0.37-1.16)  1.38(0.81-2.35)  5.65(2.69-11.86)** | 1.00  0.69(0.38-1.24)  1.43(0.80-2.57)  5.59(1.80-17.34)* |  |
| **Occupation**  Self-employed  Managers  Other white collars  Manual workers (ref)  House persons  Unemployed  Retired  Students | 70(6.7%)  65(6.2%)  178(17.1%)  214(20.5%)  53(5.1%)  85(8.2%)  329(31.6%)  48(4.6%) | 240(120-360)  360(240-480)  360(195-480)  240(120-300)  240(180-360)  300(180-390)  300(240-420)  480(300-540) | 11(15.7%)  25(38.5%)  50(28.1%)  13(6.1%)  3(5.7%)  12(14.1%)  47(14.3%)  25(52.1%) | 2.88(1.23-6.77)*  9.66(4.56-20.48)**  6.04(3.16-11.56)**  1.00  0.93(0.26-3.38)  2.54(1.11-5.82)*  2.58(1.36-4.89)*  16.81(7.57-37.29)** |  | 2.71(1.14-6.41)*  10.15(4.74-21.72)**  6.38(3.32-12.27)**  1.00  0.98(0.26-3.72)  2.51(1.08-5.8)*  1.57(0.67-3.68)  15.80(5.07-49.24)** |

*p<0.05 **p<0.001

S1 File, Table C. Sample characteristics and prevalence, univariate and multivariate odds ratio (OR) of sitting more than 7.5 hours per day, by gender, age, education and occupation, in Bulgaria. Because of co-linearity between the Education and Occupation variables we constructed two multivariate models: one including Education excluding Occupation (Model 1) and vice versa (Model 2).

| **Bulgaria** | **N (% total population)** | **Median (25^th^-75^th^ percentile) sitting minutes per day** | **N (within group %) sitting >7.5 hours per day** | **Univariate OR (95% CI) of sitting >7.5 hours per day** | **Model 1: Multivariate^b^ OR (95% CI) of sitting >7.5 hours per day** | **Model 2: Multivariate^a^ OR (95% CI) of sitting >7.5 hours per day** |
| --- | --- | --- | --- | --- | --- | --- |
| **Overall** | 947(100%) | 300(240-420) | 187(19.7%) | - | - | - |
| **Gender**  Male (ref)  Female | 436(46%)  511(54%) | 300(240-420)  300(240-420) | 83(19%)  104(20.4%) | 1.00  1.09(0.79-1.50) | 1.00  1.04(0.75-1.45) | 1.00  1.12(0.8-1.56) |
| **Age**  18-24 years  25-34 years (ref)  35-44 years  45-54 years  55-64 years  65+ years | 69(7.3%)  131(13.8%)  173(18.3%)  184(19.4%)  168(17.7%)  222(23.4%) | 300(240-420)  300(240-420)  300(180-420)  300(240-420)  360(240-420)  300(240-420) | 11(15.9%)  25(19.1%)  27(15.6%)  41(22.3%)  32(19%)  51(23%) | 0.8(0.37-1.75)  1.00  0.78(0.43-1.43)  1.22(0.70-2.12)  1(0.56-1.78)  1.26(0.74-2.16) | 0.54(0.17-1.75)  1.00  0.77(0.42-1.41)  1.14(0.65-2.01)  1.01(0.56-1.81)  1.45(0.84-2.52) | 0.68(0.2-2.29)  1.00  0.72(0.39-1.33)  1.05(0.59-1.88)  0.9(0.47-1.73)  0.98(0.42-2.29) |
| **Age stopped education**  Up to 15 years (ref)  16-19 years  20+ years  Still studying | 137(14.7%)  492(52.6%)  271(29%)  35(3.7%) | 240(180-360)  300(240-420)  360(240-420)  360(300-420) | 18(13.1%)  93(18.9%)  67(24.7%)  8(22.9%) | 1.00  1.54(0.89-2.66)  2.17(1.23-3.83)*  1.96(0.77-4.97) | 1.00  1.72(0.98-3.02)  2.46(1.36-4.45)*  4.19(1.03-16.94)* |  |
| **Occupation**  Self-employed  Managers  Other white collars  Manual workers (ref)  House persons  Unemployed  Retired  Students | 46(4.9%)  84(8.9%)  126(13.3%)  214(22.6%)  21(2.2%)  138(14.6%)  283(29.9%)  35(3.7%) | 300(180-420)  360(240-480)  360(240-480)  300(180-420)  240(180-300)  300(180-360)  300(240-420)  360(300-420) | 8(17.4%)  24(28.6%)  39(31%)  33(15.4%)  0(0%)  10(7.2%)  65(23%)  8(22.9%) | 1.15(0.49-2.7)  2.19(1.20-4.00)*  2.46(1.45-4.17)*  1.00  0(0-)  0.43(0.20-0.90)*  1.64(1.03-2.60)*  1.63(0.68-3.89) |  | 1.16(0.49-2.74)  2.20(1.2-4.02)*  2.44(1.43-4.14)*  1.00  0(0-)  0.44(0.21-0.93)*  1.50(0.72-3.13)  2.14(0.53-8.72) |

*p<0.05 **p<0.001

S1 File, Table D. Sample characteristics and prevalence, univariate and multivariate odds ratio (OR) of sitting more than 7.5 hours per day, by gender, age, education and occupation, in Croatia. Because of co-linearity between the Education and Occupation variables we constructed two multivariate models: one including Education excluding Occupation (Model 1) and vice versa (Model 2).

| **Croatia** | **N (% total population)** | **Median (25^th^-75^th^ percentile) sitting minutes per day** | **N (within group %) sitting >7.5 hours per day** | **Univariate OR (95% CI) of sitting >7.5 hours per day** | **Model 1: Multivariate^b^ OR (95% CI) of sitting >7.5 hours per day** | **Model 2: Multivariate^a^ OR (95% CI) of sitting >7.5 hours per day** |
| --- | --- | --- | --- | --- | --- | --- |
| **Overall** | 978(100%) | 300(180-420) | 222(22.7%) | - | - | - |
| **Gender**  Male (ref)  Female | 429(43.9%)  549(56.1%) | 300(180-420)  300(180-420) | 94(21.9%)  128(23.3%) | 1.00  1.08(0.80-1.47) | 1.00  1.06(0.77-1.45) | 1.00  1.04(0.75-1.43) |
| **Age**  18-24 years  25-34 years (ref)  35-44 years  45-54 years  55-64 years  65+ years | 145(14.8%)  228(23.3%)  150(15.3%)  181(18.5%)  158(16.2%)  116(11.9%) | 360(240-420)  300(180-420)  300(225-480)  270(120-420)  300(180-420)  300(180-420) | 33(22.8%)  54(23.7%)  43(28.7%)  36(19.9%)  30(19%)  26(22.4%) | 0.95(0.58-1.56)  1.00  1.3(0.81-2.07)  0.8(0.50-1.29)  0.76(0.46-1.25)  0.93(0.55-1.59) | 0.70(0.38-1.30)  1.00  1.39(0.86-2.26)  0.93(0.57-1.53)  0.83(0.49-1.40)  0.85(0.47-1.56) | 0.84(0.45-1.58)  1.00  1.39(0.84-2.29)  0.94(0.57-1.56)  1.05(0.58-1.91)  1.66(0.77-3.56) |
| **Age stopped education**  Up to 15 years (ref)  16-19 years  20+ years  Still studying | 97(10.5%)  515(55.6%)  225(24.3%)  90(9.7%) | 240(150-360)  300(180-420)  360(240-480)  360(300-480) | 13(13.4%)  99(19.2%)  72(32%)  28(31.1%) | 1.00  1.54(0.82-2.87)  3.04(1.59-5.81)*  2.92(1.40-6.09)* | 1.00  1.43(0.75-2.72)  2.77(1.43-5.39)*  3.50(1.45-8.46)* |  |
| **Occupation**  Self-employed  Managers  Other white collars  Manual workers (ref)  House persons  Unemployed  Retired  Students | 90(9.2%)  92(9.4%)  122(12.5%)  175(17.9%)  72(7.4%)  120(12.3%)  217(22.2%)  90(9.2%) | 300(180-480)  360(240-540)  360(240-540)  240(150-360)  180(120-300)  300(180-360)  240(180-390)  360(300-480) | 22(24.4%)  36(39.1%)  46(37.7%)  22(12.6%)  12(16.7%)  16(13.3%)  40(18.4%)  28(31.1%) | 2.25(1.17-4.34)*  4.47(2.42-8.25)**  4.21(2.36-7.50)**  1.00  1.39(0.65-2.99)  1.07(0.54-2.13)  1.57(0.89-2.76)  3.14(1.67-5.91)** |  | 2.10(1.08-4.08)*  4.18(2.25-7.77)**  4.20(2.34-7.53)**  1.00  1.26(0.57-2.77)  1.09(0.54-2.19)  1.23(0.59-2.57)  3.73(1.74-8.02)* |

*p<0.05 **p<0.001

S1 File, Table E. Sample characteristics and prevalence, univariate and multivariate odds ratio (OR) of sitting more than 7.5 hours per day, by gender, age, education and occupation, in the Czech Republic. Because of co-linearity between the Education and Occupation variables we constructed two multivariate models: one including Education excluding Occupation (Model 1) and vice versa (Model 2).

| **Czech Republic** | **N (% total population)** | **Median (25^th^-75^th^ percentile) sitting minutes per day** | **N (within group %) sitting >7.5 hours per day** | **Univariate OR (95% CI) of sitting >7.5 hours per day** | **Model 1: Multivariate^b^ OR (95% CI) of sitting >7.5 hours per day** | **Model 2: Multivariate^a^ OR (95% CI) of sitting >7.5 hours per day** |
| --- | --- | --- | --- | --- | --- | --- |
| **Overall** | 985(100%) | 300(180-480) | 261(26.5%) | - | - | - |
| **Gender**  Male (ref)  Female | 414(42%)  571(58%) | 300(240-420)  300(180-480) | 102(24.6%)  159(27.8%) | 1.00  1.18(0.88-1.58) | 1.00  1.28(0.95-1.73) | 1.00  1.21(0.89-1.65) |
| **Age**  18-24 years  25-34 years (ref)  35-44 years  45-54 years  55-64 years  65+ years | 82(8.3%)  158(16%)  218(22.1%)  199(20.2%)  184(18.7%)  144(14.6%) | 300(180-480)  300(180-420)  300(180-480)  300(180-480)  300(240-480)  360(240-540) | 21(25.6%)  37(23.4%)  57(26.1%)  55(27.6%)  49(26.6%)  42(29.2%) | 1.13(0.61-2.09)  1.00  1.16(0.72-1.86)  1.25(0.77-2.02)  1.19(0.73-1.94)  1.35(0.80-2.25) | 0.99(0.48-2.04)  1.00  1.33(0.82-2.17)  1.47(0.89-2.41)  1.30(0.78-2.17)  1.51(0.88-2.59) | 1.01(0.48-2.12)  1.00  1.10(0.67-1.82)  1.17(0.70-1.95)  1.25(0.69-2.26)  1.54(0.71-3.31) |
| **Age stopped education**  Up to 15 years (ref)  16-19 years  20+ years  Still studying | 39(4%)  731(74.9%)  176(18%)  30(3.1%) | 300(240-540)  300(180-420)  360(240-540)  360(300-540) | 11(28.2%)  172(23.5%)  63(35.8%)  10(33.3%) | 1.00  0.78(0.38-1.61)  1.42(0.66-3.04)  1.27(0.45-3.57) | 1.00  0.86(0.41-1.82)  1.69(0.76-3.75)  1.89(0.58-6.22) |  |
| **Occupation**  Self-employed  Managers  Other white collars  Manual workers (ref)  House persons  Unemployed  Retired  Students | 95(9.6%)  80(8.1%)  282(28.6%)  182(18.5%)  25(2.5%)  49(5%)  242(24.6%)  30(3%) | 240(180-420)  450(300-540)  360(240-480)  240(180-360)  180(120-240)  300(240-420)  360(240-480)  360(300-540) | 20(21.1%)  40(50%)  94(33.3%)  20(11%)  0(0%)  10(20.4%)  67(27.7%)  10(33.3%) | 2.16(1.10-4.25)*  8.1(4.28-15.34)**  4.05(2.39-6.86)**  1.00  0(0-)  2.08(0.90-4.79)  3.10(1.80-5.34)**  4.05(1.66-9.86)* |  | 2.25(1.13-4.44)*  8.14(4.29-15.46)**  4.05(2.39-6.88)**  1.00  0(0-)  2.03(0.87-4.76)  2.41(1.19-4.90)*  4.53(1.58-13.04)* |

*p<0.05 **p<0.001

S1 File, Table F. Sample characteristics and prevalence, univariate and multivariate odds ratio (OR) of sitting more than 7.5 hours per day, by gender, age, education and occupation, in Denmark. Because of co-linearity between the Education and Occupation variables we constructed two multivariate models: one including Education excluding Occupation (Model 1) and vice versa (Model 2).

| **Denmark** | **N (% total population)** | **Median (25^th^-75^th^ percentile) sitting minutes per day** | **N (within group %) sitting >7.5 hours per day** | **Univariate OR (95% CI) of sitting >7.5 hours per day** | **Model 1: Multivariate^b^ OR (95% CI) of sitting >7.5 hours per day** | **Model 2: Multivariate^a^ OR (95% CI) of sitting >7.5 hours per day** |
| --- | --- | --- | --- | --- | --- | --- |
| **Overall** | 984(100%) | 360(240-480) | 312(31.7%) | - | - | - |
| **Gender**  Male (ref)  Female | 483(49.1%)  501(50.9%) | 360(240-540)  360(240-480) | 166(34.4%)  146(29.1%) | 1.00  0.79(0.60-1.03) | 1.00  0.73(0.55-0.96)* | 1.00  0.74(0.56-0.98)* |
| **Age**  18-24 years  25-34 years (ref)  35-44 years  45-54 years  55-64 years  65+ years | 54(5.5%)  85(8.6%)  131(13.3%)  164(16.7%)  195(19.8%)  355(36.1%) | 420(300-540)  420(240-480)  360(240-540)  360(240-480)  360(240-480)  360(300-480) | 19(35.2%)  31(36.5%)  43(32.8%)  50(30.5%)  71(36.4%)  98(27.6%) | 0.95(0.46-1.93)  1.00  0.85(0.48-1.51)  0.76(0.44-1.33)  1(0.59-1.69)  0.66(0.40-1.09) | 0.33(0.12-0.90)*  1.00  1.00(0.54-1.83)  0.96(0.53-1.73)  1.29(0.73-2.28)  0.77(0.45-1.34) | 0.42(0.16-1.11)  1.00  1.07(0.57-2.02)  1.00(0.54-1.85)  1.31(0.72-2.40)  0.89(0.46-1.72) |
| **Age stopped education**  Up to 15 years (ref)  16-19 years  20+ years  Still studying | 42(4.4%)  155(16.3%)  699(73.6%)  54(5.7%) | 390(240-540)  360(240-480)  360(240-480)  480(405-540) | 16(38.1%)  42(27.1%)  216(30.9%)  28(51.9%) | 1.00  0.60(0.30-1.24)  0.73(0.38-1.38)  1.75(0.77-3.97) | 1.00  0.51(0.25-1.07)  0.62(0.32-1.21)  3.12(1.02-9.52)* |  |
| **Occupation**  Self-employed  Managers  Other white collars  Manual workers (ref)  House persons  Unemployed  Retired  Students | 57(5.8%)  136(13.8%)  98(10%)  194(19.7%)  4(0.4%)  37(3.8%)  404(41.1%)  54(5.5%) | 300(240-540)  420(300-540)  480(360-540)  300(180-360)  270(82.5-300)  390(240-540)  360(240-480)  480(405-540) | 22(38.6%)  53(39%)  52(53.1%)  27(13.9%)  0(0%)  15(40.5%)  115(28.5%)  28(51.9%) | 3.89(1.99-7.60)**  3.95(2.32-6.73)**  6.99(3.96-12.34)**  1.00  0(0-)  4.22(1.95-9.13)**  2.46(1.55-3.90)**  6.66(3.41-13.03)** |  | 3.61(1.83-7.14)**  3.75(2.2-6.41)**  7.20(4.05-12.77)**  1.00  0(0-)  4.80(2.18-10.59)**  2.69(1.54-4.71)*  12.83(4.87-33.77)** |

*p<0.05 **p<0.001

S1 File, Table G. Sample characteristics and prevalence, univariate and multivariate odds ratio (OR) of sitting more than 7.5 hours per day, by gender, age, education and occupation, in Estonia. Because of co-linearity between the Education and Occupation variables we constructed two multivariate models: one including Education excluding Occupation (Model 1) and vice versa (Model 2).

| **Estonia** | **N (% total population)** | **Median (25^th^-75^th^ percentile) sitting minutes per day** | **N (within group %) sitting >7.5 hours per day** | **Univariate OR (95% CI) of sitting >7.5 hours per day** | **Model 1: Multivariate^b^ OR (95% CI) of sitting >7.5 hours per day** | **Model 2: Multivariate^a^ OR (95% CI) of sitting >7.5 hours per day** |
| --- | --- | --- | --- | --- | --- | --- |
| **Overall** | 983(100%) | 300(180-420) | 224(22.8%) | - | - | - |
| **Gender**  Male (ref)  Female | 399(40.6%)  584(59.4%) | 300(180-420)  300(180-420) | 99(24.8%)  125(21.4%) | 1.00  0.83(0.61-1.12) | 1.00  0.79(0.58-1.08) | 1.00  0.80(0.58-1.11) |
| **Age**  18-24 years  25-34 years (ref)  35-44 years  45-54 years  55-64 years  65+ years | 82(8.3%)  144(14.6%)  162(16.5%)  144(14.6%)  184(18.7%)  267(27.2%) | 300(225-420)  270(180-480)  300(180-480)  300(180-420)  300(180-420)  300(240-420) | 13(15.9%)  38(26.4%)  46(28.4%)  34(23.6%)  37(20.1%)  56(21%) | 0.53(0.26-1.06)  1.00  1.11(0.67-1.83)  0.86(0.51-1.47)  0.70(0.42-1.18)  0.74(0.46-1.19) | 0.48(0.21-1.11)  1.00  1.21(0.72-2.03)  0.98(0.57-1.69)  0.78(0.46-1.34)  0.81(0.49-1.34) | 0.51(0.22-1.21)  1.00  1.17(0.69-1.99)  0.98(0.55-1.73)  0.83(0.47-1.49)  0.90(0.46-1.78) |
| **Age stopped education**  Up to 15 years (ref)  16-19 years  20+ years  Still studying | 58(6%)  442(45.7%)  425(43.9%)  43(4.4%) | 300(225-435)  240(180-420)  300(240-480)  360(180-420) | 14(24.1%)  86(19.5%)  108(25.4%)  9(20.9%) | 1.00  0.76(0.40-1.45)  1.07(0.56-2.03)  0.83(0.32-2.15) | 1.00  0.68(0.35-1.33)  0.95(0.49-1.85)  1.17(0.37-3.70) |  |
| **Occupation**  Self-employed  Managers  Other white collars  Manual workers (ref)  House persons  Unemployed  Retired  Students | 61(6.2%)  164(16.7%)  107(10.9%)  202(20.5%)  34(3.5%)  51(5.2%)  321(32.7%)  43(4.4%) | 300(195-465)  360(240-480)  360(180-540)  240(180-300)  300(180-375)  240(120-420)  300(240-420)  360(180-420) | 15(24.6%)  59(36%)  45(42.1%)  16(7.9%)  6(17.6%)  9(17.6%)  65(20.2%)  9(20.9%) | 3.79(1.75-8.23)*  6.53(3.58-11.93)**  8.44(4.45-15.98)**  1.00  2.49(0.90-6.90)  2.49(1.03-6.02)*  2.95(1.65-5.26)**  3.08(1.26-7.53)* |  | 3.70(1.70-8.07)*  6.32(3.45-11.59)**  8.48(4.46-16.12)**  1.00  2.70(0.94-7.73)  2.48(1.02-6.01)*  3.18(1.58-6.43)*  4.91(1.66-14.52)* |

*p<0.05 **p<0.001

S1 File, Table H. Sample characteristics and prevalence, univariate and multivariate odds ratio (OR) of sitting more than 7.5 hours per day, by gender, age, education and occupation, in Finland. Because of co-linearity between the Education and Occupation variables we constructed two multivariate models: one including Education excluding Occupation (Model 1) and vice versa (Model 2).

| **Finland** | **N (% total population)** | **Median (25^th^-75^th^ percentile) sitting minutes per day** | **N (within group %) sitting >7.5 hours per day** | **Univariate OR (95% CI) of sitting >7.5 hours per day** | **Model 1: Multivariate^b^ OR (95% CI) of sitting >7.5 hours per day** | **Model 2: Multivariate^a^ OR (95% CI) of sitting >7.5 hours per day** |
| --- | --- | --- | --- | --- | --- | --- |
| **Overall** | 942(100%) | 300(240-420) | 201(21.3%) | - | - | - |
| **Gender**  Male (ref)  Female | 425(45.1%)  517(54.9%) | 300(240-420)  300(240-420) | 84(19.8%)  117(22.6%) | 1.00  1.19(0.87-1.63) | 1.00  1.13(0.82-1.57) | 1.00  1.13(0.82-1.57) |
| **Age**  18-24 years  25-34 years (ref)  35-44 years  45-54 years  55-64 years  65+ years | 70(7.4%)  93(9.9%)  99(10.5%)  125(13.3%)  200(21.2%)  355(37.7%) | 360(300-420)  360(240-480)  360(240-480)  300(240-420)  300(180-420)  300(240-420) | 16(22.9%)  25(26.9%)  29(29.3%)  29(23.2%)  45(22.5%)  57(16.1%) | 0.81(0.39-1.66)  1.00  1.13(0.60-2.12)  0.82(0.44-1.53)  0.79(0.45-1.39)  0.52(0.30-0.89)* | 0.80(0.34-1.87)  1.00  1.09(0.58-2.05)  0.85(0.45-1.59)  0.84(0.47-1.51)  0.52(0.29-0.92)* | 0.96(0.41-2.22)  1.00  1.04(0.53-2.01)  0.89(0.46-1.7)  0.84(0.45-1.58)  0.52(0.25-1.08) |
| **Age stopped education**  Up to 15 years (ref)  16-19 years  20+ years  Still studying | 113(12.6%)  255(28.4%)  464(51.6%)  67(7.5%) | 300(240-420)  300(240-420)  300(240-420)  360(300-420) | 19(16.8%)  50(19.6%)  110(23.7%)  15(22.4%) | 1.00  1.21(0.67-2.16)  1.54(0.90-2.63)  1.43(0.67-3.04) | 1.00  1.02(0.56-1.85)  1.13(0.64-2.01)  0.97(0.37-2.54) |  |
| **Occupation**  Self-employed  Managers  Other white collars  Manual workers (ref)  House persons  Unemployed  Retired  Students | 74(7.9%)  93(9.9%)  65(6.9%)  172(18.3%)  15(1.6%)  57(6.1%)  399(42.4%)  67(7.1%) | 270(180-360)  420(300-540)  390(240-540)  240(180-360)  240(180-420)  360(240-420)  300(240-420)  360(300-420) | 10(13.5%)  37(39.8%)  22(33.8%)  30(17.4%)  3(20%)  12(21.1%)  72(18%)  15(22.4%) | 0.74(0.34-1.60)  3.13(1.76-5.54)**  2.42(1.27-4.63)*  1.00  1.18(0.31-4.45)  1.26(0.60-2.67)  1.04(0.65-1.67)  1.37(0.68-2.74) |  | 0.83(0.38-1.82)  3.08(1.72-5.51)**  2.34(1.22-4.50)*  1.00  1.09(0.28-4.2)  1.22(0.58-2.59)  1.57(0.86-2.89)  1.25(0.53-2.94) |

*p<0.05 **p<0.001

S1 File, Table I. Sample characteristics and prevalence, univariate and multivariate odds ratio (OR) of sitting more than 7.5 hours per day, by gender, age, education and occupation, in France. Because of co-linearity between the Education and Occupation variables we constructed two multivariate models: one including Education excluding Occupation (Model 1) and vice versa (Model 2).

| **France** | **N (% total population)** | **Median (25^th^-75^th^ percentile) sitting minutes per day** | **N (within group %) sitting >7.5 hours per day** | **Univariate OR (95% CI) of sitting >7.5 hours per day** | **Model 1: Multivariate^b^ OR (95% CI) of sitting >7.5 hours per day** | **Model 2: Multivariate^a^ OR (95% CI) of sitting >7.5 hours per day** |
| --- | --- | --- | --- | --- | --- | --- |
| **Overall** | 991(100%) | 240(180-420) | 181(18.3%) | - | - | - |
| **Gender**  Male (ref)  Female | 458(46.2%)  533(53.8%) | 270(180-420)  240(180-420) | 89(19.4%)  92(17.3%) | 1.00  0.86(0.63-1.19) | 1.00  0.83(0.59-1.16) | 1.00  0.74(0.52-1.06) |
| **Age**  18-24 years  25-34 years (ref)  35-44 years  45-54 years  55-64 years  65+ years | 92(9.3%)  145(14.6%)  142(14.3%)  169(17.1%)  176(17.8%)  267(26.9%) | 360(180-420)  240(180-360)  240(120-360)  300(180-480)  240(180-360)  300(180-420) | 22(23.9%)  22(15.2%)  21(14.8%)  46(27.2%)  23(13.1%)  47(17.6%) | 1.76(0.91-3.40)  1.00  0.97(0.51-1.86)  2.09(1.19-3.68)*  0.84(0.45-1.58)  1.19(0.69-2.08) | 0.59(0.21-1.65)  1.00  0.97(0.51-1.87)  2.28(1.28-4.04)*  0.95(0.50-1.82)  1.27(0.69-2.34) | 0.78(0.28-2.21)  1.00  0.93(0.46-1.88)  2.05(1.10-3.82)*  0.81(0.37-1.78)  1.34(0.54-3.37) |
| **Age stopped education**  Up to 15 years (ref)  16-19 years  20+ years  Still studying | 164(16.7%)  420(42.7%)  365(37.1%)  35(3.6%) | 240(180-420)  240(180-360)  300(180-420)  420(360-540) | 28(17.1%)  60(14.3%)  73(20%)  16(45.7%) | 1.00  0.81(0.50-1.32)  1.21(0.75-1.96)  4.09(1.88-8.92)** | 1.00  0.82(0.48-1.40)  1.29(0.74-2.24)  8.73(2.52-30.22)* |  |
| **Occupation**  Self-employed  Managers  Other white collars  Manual workers (ref)  House persons  Unemployed  Retired  Students | 39(3.9%)  94(9.5%)  85(8.6%)  259(26.1%)  46(4.6%)  69(7%)  364(36.7%)  35(3.5%) | 300(180-420)  300(180-435)  480(240-540)  240(120-300)  240(108.75-300)  300(180-360)  300(180-375)  420(360-540) | 7(17.9%)  23(24.5%)  44(51.8%)  19(7.3%)  1(2.2%)  11(15.9%)  60(16.5%)  16(45.7%) | 2.76(1.08-7.09)*  4.09(2.11-7.94)**  13.56(7.21-25.5)**  1.00  0.28(0.04-2.15)  2.40(1.08-5.31)*  2.49(1.45-4.29)*  10.64(4.72-23.98)** |  | 2.50(0.96-6.50)  3.99(2.04-7.82)**  15.25(7.94-29.29)**  1.00  0.37(0.05-2.87)  2.52(1.13-5.66)*  2.56(1.10-5.97)*  16.55(4.99-54.87)** |

*p<0.05 **p<0.001

S1 File, Table J. Sample characteristics and prevalence, univariate and multivariate odds ratio (OR) of sitting more than 7.5 hours per day, by gender, age, education and occupation, in Germany. Because of co-linearity between the Education and Occupation variables we constructed two multivariate models: one including Education excluding Occupation (Model 1) and vice versa (Model 2).

| **Germany** | **N (% total population)** | **Median (25^th^-75^th^ percentile) sitting minutes per day** | **N (within group %) sitting >7.5 hours per day** | **Univariate OR (95% CI) of sitting >7.5 hours per day** | **Model 1: Multivariate^b^ OR (95% CI) of sitting >7.5 hours per day** | **Model 2: Multivariate^a^ OR (95% CI) of sitting >7.5 hours per day** |
| --- | --- | --- | --- | --- | --- | --- |
| **Overall** | 1531(100%) | 300(180-420) | 280(18.3%) | - | - | - |
| **Gender**  Male (ref)  Female | 755(49.3%)  776(50.7%) | 300(180-420)  300(180-420) | 142(18.8%)  138(17.8%) | 1.00  0.93(0.72-1.21) | 1.00  0.93(0.71-1.21) | 1.00  0.92(0.70-1.22) |
| **Age**  18-24 years  25-34 years (ref)  35-44 years  45-54 years  55-64 years  65+ years | 127(8.3%)  193(12.6%)  206(13.5%)  299(19.5%)  305(19.9%)  401(26.2%) | 360(240-420)  300(180-450)  300(180-360)  240(180-360)  240(180-360)  300(180-420) | 29(22.8%)  48(24.9%)  29(14.1%)  51(17.1%)  50(16.4%)  73(18.2%) | 0.89(0.53-1.51)  1.00  0.49(0.30-0.82)*  0.62(0.40-0.97)*  0.59(0.38-0.92)*  0.67(0.44-1.02) | 0.70(0.37-1.31)  1.00  0.49(0.29-0.83)*  0.66(0.42-1.04)  0.58(0.37-0.92)*  0.61(0.39-0.95)* | 0.70(0.36-1.36)  1.00  0.53(0.31-0.91)*  0.68(0.42-1.10)  0.64(0.38-1.08)  0.73(0.36-1.46) |
| **Age stopped education**  Up to 15 years (ref)  16-19 years  20+ years  Still studying | 270(17.6%)  762(49.8%)  422(27.6%)  77(5%) | 300(180-420)  240(180-360)  300(180-420)  360(300-480) | 55(20.4%)  105(13.8%)  96(22.7%)  24(31.2%) | 1.00  0.62(0.44-0.90)*  1.15(0.79-1.67)  1.77(1.01-3.12)* | 1.00  0.59(0.40-0.87)*  1.12(0.76-1.65)  1.50(0.71-3.15) |  |
| **Occupation**  Self-employed  Managers  Other white collars  Manual workers (ref)  House persons  Unemployed  Retired  Students | 91(5.9%)  213(13.9%)  139(9.1%)  299(19.5%)  109(7.1%)  100(6.5%)  503(32.9%)  77(5%) | 300(180-420)  360(240-480)  360(240-480)  180(120-300)  240(120-300)  240(180-360)  300(180-420)  360(300-480) | 17(18.7%)  56(26.3%)  51(36.7%)  25(8.4%)  4(3.7%)  12(12%)  91(18.1%)  24(31.2%) | 2.52(1.29-4.91)*  3.91(2.35-6.51)**  6.35(3.72-10.85)**  1.00  0.42(0.14-1.23)  1.49(0.72-3.10)  2.42(1.52-3.87)**  4.96(2.64-9.34)** |  | 2.60(1.33-5.11)*  4.03(2.41-6.75)**  6.15(3.57-10.61)**  1.00  0.42(0.14-1.26)  1.48(0.71-3.07)  2.37(1.21-4.64)*  4.67(2.15-10.14)** |

*p<0.05 **p<0.001

S1 File, Table K. Sample characteristics and prevalence, univariate and multivariate odds ratio (OR) of sitting more than 7.5 hours per day, by gender, age, education and occupation, in Greece. Because of co-linearity between the Education and Occupation variables we constructed two multivariate models: one including Education excluding Occupation (Model 1) and vice versa (Model 2).

| **Greece** | **N (% total population)** | **Median (25^th^-75^th^ percentile) sitting minutes per day** | **N (within group %) sitting >7.5 hours per day** | **Univariate OR (95% CI) of sitting >7.5 hours per day** | **Model 1: Multivariate^b^ OR (95% CI) of sitting >7.5 hours per day** | **Model 2: Multivariate^a^ OR (95% CI) of sitting >7.5 hours per day** |
| --- | --- | --- | --- | --- | --- | --- |
| **Overall** | 973(100%) | 300(180-420) | 194(19.9%) | - | - | - |
| **Gender**  Male (ref)  Female | 467(48%)  506(52%) | 300(180-480)  300(180-360) | 118(25.3%)  76(15%) | 1.00  0.52(0.38-0.72)** | 1.00  0.53(0.38-0.74)** | 1.00  0.66(0.46-0.93)* |
| **Age**  18-24 years  25-34 years (ref)  35-44 years  45-54 years  55-64 years  65+ years | 86(8.8%)  161(16.5%)  199(20.5%)  176(18.1%)  151(15.5%)  200(20.6%) | 300(180-420)  240(180-360)  300(180-420)  300(180-360)  300(180-420)  360(240-480) | 19(22.1%)  18(11.2%)  44(22.1%)  27(15.3%)  25(16.6%)  61(30.5%) | 2.25(1.11-4.57)*  1.00  2.26(1.25-4.08)*  1.44(0.76-2.73)  1.58(0.82-3.02)  3.49(1.96-6.20)* | 1.87(0.75-4.71)  1.00  2.41(1.32-4.40)*  1.61(0.84-3.09)  1.77(0.89-3.52)  3.42(1.78-6.60)** | 1.79(0.71-4.52)  1.00  2.33(1.27-4.29)*  1.55(0.79-3.04)  1.91(0.91-4.02)  3.67(1.64-8.23)* |
| **Age stopped education**  Up to 15 years (ref)  16-19 years  20+ years  Still studying | 239(24.8%)  371(38.5%)  300(31.2%)  53(5.5%) | 300(240-420)  300(180-360)  300(180-420)  360(180-450) | 56(23.4%)  63(17%)  59(19.7%)  13(24.5%) | 1.00  0.67(0.45-1.00)  0.80(0.53-1.21)  1.06(0.53-2.13) | 1.00  0.83(0.52-1.34)  1.13(0.68-1.88)  1.33(0.45-3.89) |  |
| **Occupation**  Self-employed  Managers  Other white collars  Manual workers (ref)  House persons  Unemployed  Retired  Students | 177(18.2%)  40(4.1%)  117(12%)  84(8.6%)  139(14.3%)  137(14.1%)  226(23.2%)  53(5.4%) | 300(180-420)  330(180-480)  300(180-420)  240(180-360)  240(180-360)  240(180-360)  360(240-480)  360(180-450) | 32(18.1%)  14(35%)  28(23.9%)  8(9.5%)  12(8.6%)  21(15.3%)  66(29.2%)  13(24.5%) | 2.10(0.92-4.77)  5.12(1.93-13.58)*  2.99(1.29-6.95)*  1.00  0.90(0.35-2.29)  1.72(0.72-4.08)  3.92(1.79-8.57)*  3.09(1.18-8.07)* |  | 1.81(0.78-4.18)  4.92(1.83-13.24)*  3.07(1.31-7.20)*  1.00  0.89(0.32-2.44)  1.75(0.73-4.21)  2.10(0.81-5.45)  2.84(0.84-9.57) |

*p<0.05 **p<0.001

S1 File, Table L. Sample characteristics and prevalence, univariate and multivariate odds ratio (OR) of sitting more than 7.5 hours per day, by gender, age, education and occupation, in Hungary. Because of co-linearity between the Education and Occupation variables we constructed two multivariate models: one including Education excluding Occupation (Model 1) and vice versa (Model 2).

| **Hungary** | **N (% total population)** | **Median (25^th^-75^th^ percentile) sitting minutes per day** | **N (within group %) sitting >7.5 hours per day** | **Univariate OR (95% CI) of sitting >7.5 hours per day** | **Model 1: Multivariate^b^ OR (95% CI) of sitting >7.5 hours per day** | **Model 2: Multivariate^a^ OR (95% CI) of sitting >7.5 hours per day** |
| --- | --- | --- | --- | --- | --- | --- |
| **Overall** | 974(100%) | 240(120-360) | 115(11.8%) | - | - | - |
| **Gender**  Male (ref)  Female | 402(41.3%)  572(58.7%) | 240(120-300)  240(180-360) | 46(11.4%)  69(12.1%) | 1.00  1.06(0.71-1.58) | 1.00  0.99(0.66-1.49) | 1.00  0.99(0.65-1.5) |
| **Age**  18-24 years  25-34 years (ref)  35-44 years  45-54 years  55-64 years  65+ years | 75(7.7%)  160(16.4%)  204(20.9%)  148(15.2%)  195(20%)  192(19.7%) | 180(120-360)  240(120-300)  180(120-300)  240(180-360)  240(180-360)  300(180-360) | 9(12%)  15(9.4%)  19(9.3%)  21(14.2%)  25(12.8%)  26(13.5%) | 1.32(0.55-3.17)  1.00  0.99(0.49-2.02)  1.60(0.79-3.23)  1.42(0.72-2.80)  1.51(0.77-2.97) | 0.76(0.23-2.56)  1.00  1.08(0.53-2.22)  1.73(0.85-3.52)  1.51(0.76-3.00)  1.53(0.76-3.09) | 0.66(0.19-2.24)  1.00  0.94(0.46-1.94)  1.53(0.75-3.15)  1.65(0.75-3.65)  2.01(0.78-5.2) |
| **Age stopped education**  Up to 15 years (ref)  16-19 years  20+ years  Still studying | 195(20.3%)  585(61%)  150(15.6%)  29(3%) | 240(180-360)  240(120-300)  240(165-360)  300(180-420) | 29(14.9%)  57(9.7%)  23(15.3%)  6(20.7%) | 1.00  0.62(0.38-1.00)*  1.04(0.57-1.88)  1.49(0.56-3.98) | 1.00  0.67(0.40-1.10)  1.17(0.63-2.18)  2.74(0.64-11.68) |  |
| **Occupation**  Self-employed  Managers  Other white collars  Manual workers (ref)  House persons  Unemployed  Retired  Students | 48(4.9%)  44(4.5%)  134(13.8%)  273(28%)  38(3.9%)  81(8.3%)  327(33.6%)  29(3%) | 180(108.75-300)  240(120-360)  300(120-420)  180(120-300)  180(120-240)  210(120-300)  240(180-360)  300(180-420) | 7(14.6%)  5(11.4%)  24(17.9%)  24(8.8%)  4(10.5%)  5(6.2%)  40(12.2%)  6(20.7%) | 1.77(0.72-4.38)  1.33(0.48-3.69)  2.26(1.23-4.16)*  1.00  1.22(0.40-3.73)  0.68(0.25-1.85)  1.45(0.85-2.47)  2.71(1.00-7.29)* |  | 1.57(0.63-3.92)  1.32(0.47-3.69)  2.28(1.22-4.25)*  1.00  1.39(0.44-4.41)  0.72(0.26-1.99)  0.91(0.42-1.98)  4.66(1.11-19.68)* |

*p<0.05 **p<0.001

S1 File, Table M. Sample characteristics and prevalence, univariate and multivariate odds ratio (OR) of sitting more than 7.5 hours per day, by gender, age, education and occupation, in Ireland. Because of co-linearity between the Education and Occupation variables we constructed two multivariate models: one including Education excluding Occupation (Model 1) and vice versa (Model 2).

| **Ireland** | **N (% total population)** | **Median (25^th^-75^th^ percentile) sitting minutes per day** | **N (within group %) sitting >7.5 hours per day** | **Univariate OR (95% CI) of sitting >7.5 hours per day** | **Model 1: Multivariate^b^ OR (95% CI) of sitting >7.5 hours per day** | **Model 2: Multivariate^a^ OR (95% CI) of sitting >7.5 hours per day** |
| --- | --- | --- | --- | --- | --- | --- |
| **Overall** | 953(100%) | 240(180-360) | 99(10.4%) | - | - | - |
| **Gender**  Male (ref)  Female | 418(43.9%)  535(56.1%) | 300(180-360)  240(180-300) | 52(12.4%)  47(8.8%) | 1.00  0.68(0.45-1.03) | 1.00  0.68(0.45-1.04) | 1.00  0.69(0.43-1.10) |
| **Age**  18-24 years  25-34 years (ref)  35-44 years  45-54 years  55-64 years  65+ years | 67(7%)  183(19.2%)  210(22%)  143(15%)  159(16.7%)  191(20%) | 240(180-360)  240(120-300)  240(120-360)  240(180-300)  300(180-360)  300(240-360) | 7(10.4%)  15(8.2%)  23(11%)  13(9.1%)  21(13.2%)  20(10.5%) | 1.31(0.51-3.36)  1.00  1.38(0.70-2.73)  1.12(0.51-2.44)  1.70(0.85-3.43)  1.31(0.65-2.64) | 0.54(0.13-2.24)  1.00  1.37(0.68-2.74)  1.36(0.61-3.03)  2.01(0.96-4.20)  1.59(0.74-3.43) | 0.56(0.13-2.43)  1.00  1.24(0.61-2.5)  1.17(0.53-2.61)  1.98(0.93-4.2)  1.6(0.67-3.79) |
| **Age stopped education**  Up to 15 years (ref)  16-19 years  20+ years  Still studying | 174(18.5%)  446(47.3%)  276(29.3%)  46(4.9%) | 300(180-360)  240(180-360)  240(180-360)  300(225-420) | 19(10.9%)  36(8.1%)  35(12.7%)  8(17.4%) | 1.00  0.72(0.40-1.29)  1.18(0.65-2.15)  1.72(0.70-4.22) | 1.00  0.85(0.45-1.58)  1.53(0.78-3.02)  4.44(1.06-18.62)* |  |
| **Occupation**  Self-employed  Managers  Other white collars  Manual workers (ref)  House persons  Unemployed  Retired  Students | 84(8.8%)  95(10%)  91(9.5%)  193(20.3%)  216(22.7%)  86(9%)  142(14.9%)  46(4.8%) | 240(180-330)  240(180-390)  300(180-420)  240(120-300)  240(180-300)  240(180-360)  300(240-360)  300(225-420) | 8(9.5%)  17(17.9%)  19(20.9%)  10(5.2%)  14(6.5%)  6(7%)  17(12%)  8(17.4%) | 1.93(0.73-5.07)  3.99(1.75-9.10)*  4.83(2.14-10.89)**  1.00  1.27(0.55-2.93)  1.37(0.48-3.91)  2.49(1.10-5.61)*  3.85(1.43-10.40)* |  | 1.61(0.6-4.32)  4.28(1.85-9.9)*  4.86(2.13-11.05)**  1.00  1.29(0.52-3.17)  1.34(0.47-3.84)  1.74(0.67-4.49)  7.38(1.71-31.82)* |

*p<0.05 **p<0.001

S1 File, Table N. Sample characteristics and prevalence, univariate and multivariate odds ratio (OR) of sitting more than 7.5 hours per day, by gender, age, education and occupation, in Italy. Because of co-linearity between the Education and Occupation variables we constructed two multivariate models: one including Education excluding Occupation (Model 1) and vice versa (Model 2).

| **Italy** | **N (% total population)** | **Median (25^th^-75^th^ percentile) sitting minutes per day** | **N (within group %) sitting >7.5 hours per day** | **Univariate OR (95% CI) of sitting >7.5 hours per day** | **Model 1: Multivariate^b^ OR (95% CI) of sitting >7.5 hours per day** | **Model 2: Multivariate^a^ OR (95% CI) of sitting >7.5 hours per day** |
| --- | --- | --- | --- | --- | --- | --- |
| **Overall** | 955(100%) | 240(180-360) | 105(11%) | - | - | - |
| **Gender**  Male (ref)  Female | 424(44.4%)  531(55.6%) | 240(180-360)  240(180-360) | 55(13%)  50(9.4%) | 1.00  0.70(0.46-1.05) | 1.00  0.71(0.47-1.08) | 1.00  0.66(0.42-1.04) |
| **Age**  18-24 years  25-34 years (ref)  35-44 years  45-54 years  55-64 years  65+ years | 74(7.7%)  132(13.8%)  226(23.7%)  221(23.1%)  152(15.9%)  150(15.7%) | 300(240-360)  240(180-360)  240(180-360)  240(120-360)  240(120-330)  300(180-360) | 7(9.5%)  16(12.1%)  25(11.1%)  24(10.9%)  15(9.9%)  18(12%) | 0.76(0.30-1.93)  1.00  0.9(0.46-1.76)  0.88(0.45-1.73)  0.79(0.38-1.67)  0.99(0.48-2.03) | 0.66(0.20-2.19)  1.00  0.87(0.44-1.73)  0.96(0.48-1.90)  0.96(0.44-2.07)  1.26(0.57-2.77) | 0.96(0.25-3.60)  1.00  0.92(0.45-1.86)  0.99(0.49-2.03)  1.33(0.57-3.07)  2.44(0.77-7.77) |
| **Age stopped education**  Up to 15 years (ref)  16-19 years  20+ years  Still studying | 292(31.3%)  393(42.1%)  191(20.4%)  58(6.2%) | 240(120-300)  240(180-360)  300(180-360)  300(240-360) | 20(6.8%)  47(12%)  29(15.2%)  6(10.3%) | 1.00  1.85(1.07-3.19)*  2.43(1.33-4.44)*  1.57(0.60-4.10) | 1.00  1.98(1.11-3.54)*  2.62(1.37-5.00)*  2.20(0.57-8.50) |  |
| **Occupation**  Self-employed  Managers  Other white collars  Manual workers (ref)  House persons  Unemployed  Retired  Students | 131(13.7%)  58(6.1%)  192(20.1%)  163(17.1%)  143(15%)  42(4.4%)  168(17.6%)  58(6.1%) | 240(120-360)  240(120-300)  360(180-465)  240(120-300)  240(120-300)  180(120-300)  300(180-360)  300(240-360) | 10(7.6%)  5(8.6%)  48(25%)  9(5.5%)  7(4.9%)  2(4.8%)  18(10.7%)  6(10.3%) | 1.41(0.56-3.59)  1.61(0.52-5.03)  5.70(2.70-12.04)**  1.00  0.88(0.32-2.43)  0.86(0.18-4.12)  2.05(0.89-4.71)  1.97(0.67-5.81) |  | 1.30(0.51-3.32)  1.74(0.56-5.47)  6.41(3.00-13.71)**  1.00  0.97(0.33-2.87)  0.93(0.19-4.60)  1.01(0.31-3.28)  2.19(0.49-9.78) |

*p<0.05 **p<0.001

S1 File, Table O. Sample characteristics and prevalence, univariate and multivariate odds ratio (OR) of sitting more than 7.5 hours per day, by gender, age, education and occupation in Latvia. Because of co-linearity between the Education and Occupation variables we constructed two multivariate models: one including Education excluding Occupation (Model 1) and vice versa (Model 2).

| **Latvia** | **N (% total population)** | **Median (25^th^-75^th^ percentile) sitting minutes per day** | **N (within group %) sitting >7.5 hours per day** | **Univariate OR (95% CI) of sitting >7.5 hours per day** | **Model 1: Multivariate^b^ OR (95% CI) of sitting >7.5 hours per day** | **Model 2: Multivariate^a^ OR (95% CI) of sitting >7.5 hours per day** |
| --- | --- | --- | --- | --- | --- | --- |
| **Overall** | 964(100%) | 300(180-420) | 168(17.4%) | - | - | - |
| **Gender**  Male (ref)  Female | 437(45.3%)  527(54.7%) | 240(180-360)  300(180-420) | 58(13.3%)  110(20.9%) | 1.00  1.72(1.22-2.44)* | 1.00  1.71(1.20-2.44)* | 1.00  1.62(1.11-2.36)* |
| **Age**  18-24 years  25-34 years (ref)  35-44 years  45-54 years  55-64 years  65+ years | 147(15.2%)  177(18.4%)  172(17.8%)  192(19.9%)  134(13.9%)  142(14.7%) | 300(180-420)  300(180-420)  300(180-405)  300(180-360)  240(180-360)  300(180-420) | 28(19%)  32(18.1%)  31(18%)  33(17.2%)  14(10.4%)  30(21.1%) | 1.07(0.61-1.87)  1.00  1.00(0.58-1.72)  0.94(0.55-1.61)  0.53(0.27-1.04)  1.21(0.70-2.12) | 0.81(0.43-1.56)  1.00  1.12(0.64-1.95)  1.03(0.60-1.78)  0.57(0.29-1.13)  1.18(0.66-2.08) | 0.89(0.45-1.75)  1.00  1.05(0.58-1.88)  0.94(0.53-1.68)  0.50(0.24-1.03)  0.99(0.43-2.31) |
| **Age stopped education**  Up to 15 years (ref)  16-19 years  20+ years  Still studying | 47(4.9%)  515(53.6%)  339(35.3%)  60(6.2%) | 240(180-480)  240(180-360)  300(180-420)  360(300-480) | 12(25.5%)  76(14.8%)  61(18%)  19(31.7%) | 1.00  0.51(0.25-1.02)  0.64(0.31-1.30)  1.35(0.58-3.17) | 1.00  0.52(0.25-1.07)  0.65(0.31-1.35)  1.61(0.59-4.34) |  |
| **Occupation**  Self-employed  Managers  Other white collars  Manual workers (ref)  House persons  Unemployed  Retired  Students | 71(7.4%)  114(11.8%)  97(10.1%)  292(30.3%)  44(4.6%)  99(10.3%)  187(19.4%)  60(6.2%) | 300(180-420)  300(180-420)  420(180-480)  240(120-300)  240(120-300)  240(180-360)  300(180-420)  360(300-480) | 15(21.1%)  26(22.8%)  38(39.2%)  19(6.5%)  4(9.1%)  12(12.1%)  35(18.7%)  19(31.7%) | 3.85(1.85-8.03)**  4.25(2.24-8.04)**  9.25(4.99-17.18)**  1.00  1.44(0.47-4.44)  1.98(0.93-4.25)  3.31(1.83-5.99)**  6.66(3.26-13.62)** |  | 4.00(1.89-8.44)**  3.67(1.92-7.02)**  8.89(4.76-16.6)**  1.00  1.19(0.38-3.72)  1.86(0.86-4.02)  3.08(1.37-6.92)*  6.35(2.72-14.78)** |

*p<0.05 **p<0.001

S1 File, Table P. Sample characteristics and prevalence, univariate and multivariate odds ratio (OR) of sitting more than 7.5 hours per day, by gender, age, education and occupation, in Lithuania. Because of co-linearity between the Education and Occupation variables we constructed two multivariate models: one including Education excluding Occupation (Model 1) and vice versa (Model 2).

| **Lithuania** | **N (% total population)** | **Median (25^th^-75^th^ percentile) sitting minutes per day** | **N (within group %) sitting >7.5 hours per day** | **Univariate OR (95% CI) of sitting >7.5 hours per day** | **Model 1: Multivariate^b^ OR (95% CI) of sitting >7.5 hours per day** | **Model 2: Multivariate^a^ OR (95% CI) of sitting >7.5 hours per day** |
| --- | --- | --- | --- | --- | --- | --- |
| **Overall** | 963(100%) | 300(180-420) | 161(16.7%) | - | - | - |
| **Gender**  Male (ref)  Female | 431(44.8%)  532(55.2%) | 240(180-360)  300(180-420) | 69(16%)  92(17.3%) | 1.00  1.10(0.78-1.54) | 1.00  1.07(0.75-1.53) | 1.00  1.10(0.77-1.58) |
| **Age**  18-24 years  25-34 years (ref)  35-44 years  45-54 years  55-64 years  65+ years | 137(14.2%)  133(13.8%)  154(16%)  223(23.2%)  118(12.3%)  198(20.6%) | 300(240-420)  300(225-360)  240(180-360)  240(180-360)  240(180-360)  300(180-435) | 23(16.8%)  17(12.8%)  24(15.6%)  33(14.8%)  15(12.7%)  49(24.7%) | 1.38(0.70-2.71)  1.00  1.26(0.65-2.46)  1.19(0.63-2.22)  0.99(0.47-2.09)  2.24(1.23-4.10)* | 0.72(0.28-1.84)  1.00  1.39(0.70-2.76)  1.28(0.67-2.42)  1.02(0.48-2.19)  2.28(1.20-4.34)* | 1.02(0.41-2.53)  1.00  1.44(0.71-2.91)  1.51(0.77-2.95)  0.83(0.35-1.95)  1.48(0.63-3.50) |
| **Age stopped education**  Up to 15 years (ref)  16-19 years  20+ years  Still studying | 58(6.2%)  458(49.3%)  345(37.1%)  68(7.3%) | 330(180-480)  240(180-360)  300(180-420)  360(300-420) | 15(25.9%)  59(12.9%)  64(18.6%)  16(23.5%) | 1.00  0.42(0.22-0.81)*  0.65(0.34-1.25)  0.88(0.39-1.99) | 1.00  0.65(0.32-1.31)  1.05(0.52-2.13)  2.46(0.78-7.80) |  |
| **Occupation**  Self-employed  Managers  Other white collars  Manual workers (ref)  House persons  Unemployed  Retired  Students | 41(4.3%)  103(10.7%)  93(9.7%)  238(24.7%)  35(3.6%)  106(11%)  279(29%)  68(7.1%) | 180(180-240)  300(240-435)  300(180-480)  240(180-300)  240(180-300)  300(180-360)  300(180-420)  360(300-420) | 2(4.9%)  25(24.3%)  25(26.9%)  15(6.3%)  3(8.6%)  10(9.4%)  65(23.3%)  16(23.5%) | 0.76(0.17-3.47)  4.77(2.39-9.50)**  5.47(2.73-10.96)**  1.00  1.39(0.38-5.08)  1.55(0.67-3.57)  4.52(2.50-8.16)**  4.57(2.13-9.84)** |  | 0.74(0.16-3.36)  5.21(2.57-10.54)**  5.64(2.80-11.35)**  1.00  1.51(0.41-5.63)  1.59(0.69-3.69)  4.36(2.01-9.49)**  5.69(2.06-15.73)* |

*p<0.05 **p<0.001

S1 File, Table Q. Sample characteristics and prevalence, univariate and multivariate odds ratio (OR) of sitting more than 7.5 hours per day, by gender, age, education and occupation, in Luxembourg. Because of co-linearity between the Education and Occupation variables we constructed two multivariate models: one including Education excluding Occupation (Model 1) and vice versa (Model 2).

| **Luxembourg** | **N (% total population)** | **Median (25^th^-75^th^ percentile) sitting minutes per day** | **N (within group %) sitting >7.5 hours per day** | **Univariate OR (95% CI) of sitting >7.5 hours per day** | **Model 1: Multivariate^b^ OR (95% CI) of sitting >7.5 hours per day** | **Model 2: Multivariate^a^ OR (95% CI) of sitting >7.5 hours per day** |
| --- | --- | --- | --- | --- | --- | --- |
| **Overall** | 484(100%) | 300(180-420) | 100(20.7%) | - | - | - |
| **Gender**  Male (ref)  Female | 196(40.5%)  288(59.5%) | 300(240-480)  240(180-420) | 54(27.6%)  46(16%) | 1.00  0.50(0.32-0.78)* | 1.00  0.49(0.31-0.79)* | 1.00  0.57(0.34-0.95)* |
| **Age**  18-24 years  25-34 years (ref)  35-44 years  45-54 years  55-64 years  65+ years | 13(2.7%)  78(16.1%)  71(14.7%)  100(20.7%)  89(18.4%)  133(27.5%) | 480(180-540)  240(120-420)  300(180-480)  300(180-420)  300(180-360)  300(240-420) | 7(53.8%)  15(19.2%)  22(31%)  24(24%)  10(11.2%)  22(16.5%) | 4.90(1.44-16.72)*  1.00  1.89(0.89-4.01)  1.33(0.64-2.74)  0.53(0.22-1.26)  0.83(0.40-1.72) | 5.08(0.82-31.67)  1.00  2.22(1.01-4.87)*  1.63(0.77-3.44)  0.67(0.28-1.62)  1.09(0.51-2.34) | 6.42(0.8-51.41)  1.00  2.04(0.84-4.96)  1.15(0.5-2.64)  0.56(0.2-1.59)  0.98(0.33-2.98) |
| **Age stopped education**  Up to 15 years (ref)  16-19 years  20+ years  Still studying | 104(21.8%)  147(30.8%)  215(45.1%)  11(2.3%) | 240(135-345)  300(180-360)  360(240-480)  480(180-540) | 9(8.7%)  25(17%)  58(27%)  6(54.5%) | 1.00  2.16(0.96-4.85)  3.90(1.85-8.23)**  12.67(3.22-49.83)** | 1.00  2.25(0.98-5.14)  3.87(1.78-8.41)*  4.25(0.59-30.61) |  |
| **Occupation**  Self-employed  Managers  Other white collars  Manual workers (ref)  House persons  Unemployed  Retired  Students | 27(5.6%)  48(9.9%)  58(12%)  106(21.9%)  44(9.1%)  15(3.1%)  175(36.2%)  11(2.3%) | 240(180-420)  420(300-540)  450(300-540)  180(120-240)  240(180-360)  420(285-480)  300(240-360)  480(180-540) | 2(7.4%)  22(45.8%)  29(50%)  6(5.7%)  2(4.5%)  5(33.3%)  28(16%)  6(54.5%) | 1.33(0.25-7.01)  14.10(5.19-38.35)**  16.67(6.31-44.03)**  1.00  0.79(0.15-4.09)  8.33(2.15-32.25)*  3.17(1.27-7.95)*  20.00(4.72-84.81)** |  | 1.79(0.33-9.72)  14.19(5-40.28)**  20.18(7.32-55.63)**  1.00  1.3(0.24-7.08)  8.37(2.05-34.09)*  4.77(1.47-15.47)*  5.91(0.72-48.58) |

*p<0.05 **p<0.001

S1 File, Table R. Sample characteristics and prevalence, univariate and multivariate odds ratio (OR) of sitting more than 7.5 hours per day, by gender, age, education and occupation, in Malta. Because of co-linearity between the Education and Occupation variables we constructed two multivariate models: one including Education excluding Occupation (Model 1) and vice versa (Model 2).

| **Malta** | **N (% total population)** | **Median (25^th^-75^th^ percentile) sitting minutes per day** | **N (within group %) sitting >7.5 hours per day** | **Univariate OR (95% CI) of sitting >7.5 hours per day** | **Model 1: Multivariate^b^ OR (95% CI) of sitting >7.5 hours per day** | **Model 2: Multivariate^a^ OR (95% CI) of sitting >7.5 hours per day** |
| --- | --- | --- | --- | --- | --- | --- |
| **Overall** | 488(100%) | 240(120-360) | 58(11.9%) | - | - | - |
| **Gender**  Male (ref)  Female | 197(40.4%)  291(59.6%) | 240(120-360)  180(120-300) | 29(14.7%)  29(10%) | 1.00  0.64(0.37-1.11) | 1.00  0.74(0.41-1.35) | 1.00  1.19(0.60-2.35) |
| **Age**  18-24 years  25-34 years (ref)  35-44 years  45-54 years  55-64 years  65+ years | 33(6.8%)  49(10%)  42(8.6%)  70(14.3%)  122(25%)  172(35.2%) | 300(135-480)  240(120-450)  180(75-360)  180(75-255)  180(75-255)  240(180-360) | 10(30.3%)  12(24.5%)  5(11.9%)  6(8.6%)  11(9%)  14(8.1%) | 1.34(0.50-3.60)  1.00  0.42(0.13-1.30)  0.29(0.10-0.83)*  0.31(0.12-0.75)*  0.27(0.12-0.64)* | 1.14(0.38-3.46)  1.00  0.40(0.12-1.31)  0.29(0.10-0.86)*  0.44(0.17-1.13)  0.33(0.13-0.83)* | 1.04(0.32-3.35)  1.00  0.68(0.20-2.35)  0.52(0.16-1.64)  1.08(0.37-3.15)  1.49(0.43-5.16) |
| **Age stopped education**  Up to 15 years (ref)  16-19 years  20+ years  Still studying | 176(36.7%)  225(47%)  65(13.6%)  13(2.7%) | 180(120-300)  240(120-360)  240(180-390)  360(240-510) | 9(5.1%)  31(13.8%)  11(16.9%)  5(38.5%) | 1.00  2.97(1.37-6.41)*  3.78(1.49-9.61)*  11.6(3.15-42.69)** | 1.00  2.58(1.12-5.94)*  2.45(0.87-6.86)  3.91(0.79-19.33) |  |
| **Occupation**  Self-employed  Managers  Other white collars  Manual workers (ref)  House persons  Unemployed  Retired  Students | 22(4.5%)  39(8%)  37(7.6%)  59(12.1%)  192(39.3%)  9(1.8%)  117(24%)  13(2.7%) | 210(108.75-480)  300(180-420)  420(240-510)  120(75-240)  180(120-255)  240(150-360)  240(180-360)  360(240-510) | 6(27.3%)  9(23.1%)  16(43.2%)  5(8.5%)  5(2.6%)  1(11.1%)  11(9.4%)  5(38.5%) | 4.05(1.09-15.03)*  3.24(0.99-10.55)  8.23(2.68-25.31)**  1.00  0.29(0.08-1.03)  1.35(0.14-13.09)  1.12(0.37-3.39)  6.75(1.59-28.63)* |  | 3.49(0.90-13.53)  3.12(0.93-10.38)  7.55(2.41-23.69)*  1.00  0.19(0.04-0.83)*  1.48(0.15-14.72)  0.70(0.18-2.73)  5.32(1.02-27.71)* |

*p<0.05 **p<0.001

S1 File, Table S. Sample characteristics and prevalence, univariate and multivariate odds ratio (OR) of sitting more than 7.5 hours per day, by gender, age, education and occupation, in the Netherlands. Because of co-linearity between the Education and Occupation variables we constructed two multivariate models: one including Education excluding Occupation (Model 1) and vice versa (Model 2).

| **Netherlands** | **N (% total population)** | **Median (25^th^-75^th^ percentile) sitting minutes per day** | **N (within group %) sitting >7.5 hours per day** | **Univariate OR (95% CI) of sitting >7.5 hours per day** | **Model 1: Multivariate^b^ OR (95% CI) of sitting >7.5 hours per day** | **Model 2: Multivariate^a^ OR (95% CI) of sitting >7.5 hours per day** |
| --- | --- | --- | --- | --- | --- | --- |
| **Overall** | 991(100%) | 360(240-480) | 318(32.1%) | - | - | - |
| **Gender**  Male (ref)  Female | 451(45.5%)  540(54.5%) | 420(300-540)  360(240-480) | 166(36.8%)  152(28.1%) | 1.00  0.67(0.51-0.88)* | 1.00  0.69(0.53-0.91)* | 1.00  0.79(0.59-1.05) |
| **Age**  18-24 years  25-34 years (ref)  35-44 years  45-54 years  55-64 years  65+ years | 61(6.2%)  108(10.9%)  120(12.1%)  180(18.2%)  231(23.3%)  291(29.4%) | 360(300-480)  360(240-540)  360(240-540)  360(300-480)  360(240-480)  360(300-480) | 18(29.5%)  37(34.3%)  42(35%)  59(32.8%)  74(32%)  88(30.2%) | 0.80(0.41-1.58)  1.00  1.03(0.60-1.79)  0.94(0.57-1.55)  0.90(0.56-1.47)  0.83(0.52-1.33) | 0.55(0.22-1.39)  1.00  1.07(0.62-1.86)  1.01(0.60-1.70)  0.97(0.59-1.60)  0.93(0.57-1.53) | 0.76(0.31-1.82)  1.00  1.16(0.65-2.07)  1.05(0.61-1.8)  0.99(0.58-1.69)  0.79(0.41-1.55) |
| **Age stopped education**  Up to 15 years (ref)  16-19 years  20+ years  Still studying | 96(9.8%)  347(35.3%)  499(50.8%)  40(4.1%) | 360(255-480)  360(240-480)  360(300-540)  390(300-525) | 28(29.2%)  92(26.5%)  179(35.9%)  13(32.5%) | 1.00  0.88(0.53-1.45)  1.36(0.84-2.19)  1.17(0.53-2.59) | 1.00  0.84(0.50-1.41)  1.22(0.74-2.03)  1.60(0.54-4.71) |  |
| **Occupation**  Self-employed  Managers  Other white collars  Manual workers (ref)  House persons  Unemployed  Retired  Students | 102(10.3%)  132(13.3%)  143(14.4%)  131(13.2%)  68(6.9%)  56(5.7%)  319(32.2%)  40(4%) | 300(240-480)  420(300-480)  480(360-540)  300(180-360)  300(180-420)  360(240-480)  360(300-480)  390(300-525) | 28(27.5%)  48(36.4%)  77(53.8%)  15(11.5%)  11(16.2%)  19(33.9%)  107(33.5%)  13(32.5%) | 2.93(1.47-5.84)*  4.42(2.32-8.42)**  9.02(4.80-16.95)**  1.00  1.49(0.64-3.46)  3.97(1.84-8.59)**  3.90(2.17-7.01)**  3.72(1.59-8.74)* |  | 2.78(1.38-5.59)*  4.2(2.2-8.02)**  8.6(4.56-16.21)**  1.00  1.72(0.73-4.07)  3.86(1.77-8.39)*  4.54(2.25-9.17)**  4.37(1.52-12.57)* |

*p<0.05 **p<0.001

S1 File, Table T. Sample characteristics and prevalence, univariate and multivariate odds ratio (OR) of sitting more than 7.5 hours per day, by gender, age, education and occupation, in Poland. Because of co-linearity between the Education and Occupation variables we constructed two multivariate models: one including Education excluding Occupation (Model 1) and vice versa (Model 2).

| **Poland** | **N (% total population)** | **Median (25^th^-75^th^ percentile) sitting minutes per day** | **N (within group %) sitting >7.5 hours per day** | **Univariate OR (95% CI) of sitting >7.5 hours per day** | **Model 1: Multivariate^b^ OR (95% CI) of sitting >7.5 hours per day** | **Model 2: Multivariate^a^ OR (95% CI) of sitting >7.5 hours per day** |
| --- | --- | --- | --- | --- | --- | --- |
| **Overall** | 879(100%) | 240(180-360) | 158(18%) | - | - | - |
| **Gender**  Male (ref)  Female | 337(38.3%)  542(61.7%) | 240(180-360)  240(120-390) | 56(16.6%)  102(18.8%) | 1.00  1.16(0.81-1.67) | 1.00  1.18(0.81-1.72) | 1.00  1.05(0.71-1.55) |
| **Age**  18-24 years  25-34 years (ref)  35-44 years  45-54 years  55-64 years  65+ years | 81(9.2%)  146(16.6%)  152(17.3%)  145(16.5%)  188(21.4%)  167(19%) | 270(180-420)  300(180-420)  240(180-420)  240(120-360)  240(120-360)  300(180-405) | 17(21%)  34(23.3%)  32(21.1%)  24(16.6%)  20(10.6%)  31(18.6%) | 0.88(0.45-1.69)  1.00  0.88(0.51-1.52)  0.65(0.37-1.17)  0.39(0.22-0.72)*  0.75(0.43-1.30) | 1.08(0.47-2.47)  1.00  1.01(0.57-1.78)  0.76(0.41-1.40)  0.45(0.24-0.87)*  0.87(0.47-1.63) | 1.41(0.58-3.41)  1.00  0.94(0.52-1.69)  0.76(0.41-1.43)  0.58(0.28-1.18)  1.35(0.57-3.20) |
| **Age stopped education**  Up to 15 years (ref)  16-19 years  20+ years  Still studying | 91(10.6%)  452(52.7%)  280(32.7%)  34(4%) | 240(180-360)  240(120-360)  300(180-480)  300(180-435) | 16(17.6%)  55(12.2%)  71(25.4%)  8(23.5%) | 1.00  0.65(0.35-1.19)  1.59(0.87-2.91)  1.44(0.55-3.76) | 1.00  0.63(0.32-1.23)  1.44(0.73-2.83)  1.02(0.30-3.46) |  |
| **Occupation**  Self-employed  Managers  Other white collars  Manual workers (ref)  House persons  Unemployed  Retired  Students | 72(8.2%)  83(9.4%)  108(12.3%)  175(19.9%)  69(7.8%)  59(6.7%)  279(31.7%)  34(3.9%) | 240(120-360)  360(240-480)  360(180-480)  240(120-300)  240(120-300)  240(120-360)  240(180-360)  300(180-435) | 12(16.7%)  28(33.7%)  44(40.7%)  17(9.7%)  5(7.2%)  5(8.5%)  39(14%)  8(23.5%) | 1.86(0.84-4.12)  4.73(2.41-9.31)**  6.39(3.40-12.00)**  1.00  0.73(0.26-2.05)  0.86(0.30-2.44)  1.51(0.83-2.76)  2.86(1.12-7.30)* |  | 1.93(0.86-4.33)  4.80(2.40-9.59)**  6.27(3.26-12.07)**  1.00  0.68(0.24-1.99)  0.81(0.28-2.34)  1.32(0.56-3.10)  1.81(0.56-5.83) |

*p<0.05 **p<0.001

S1 File, Table U. Sample characteristics and prevalence, univariate and multivariate odds ratio (OR) of sitting more than 7.5 hours per day, by gender, age, education and occupation, in Portugal. Because of co-linearity between the Education and Occupation variables we constructed two multivariate models: one including Education excluding Occupation (Model 1) and vice versa (Model 2).

| **Portugal** | **N (% total population)** | **Median (25^th^-75^th^ percentile) sitting minutes per day** | **N (within group %) sitting >7.5 hours per day** | **Univariate OR (95% CI) of sitting >7.5 hours per day** | **Model 1: Multivariate^b^ OR (95% CI) of sitting >7.5 hours per day** | **Model 2: Multivariate^a^ OR (95% CI) of sitting >7.5 hours per day** |
| --- | --- | --- | --- | --- | --- | --- |
| **Overall** | 980(100%) | 180(120-360) | 98(10%) | - | - | - |
| **Gender**  Male (ref)  Female | 441(45%)  539(55%) | 240(120-360)  180(120-300) | 51(11.6%)  47(8.7%) | 1.00  0.73(0.48-1.11) | 1.00  0.78(0.50-1.21) | 1.00  0.77(0.49-1.20) |
| **Age**  18-24 years  25-34 years (ref)  35-44 years  45-54 years  55-64 years  65+ years | 82(8.4%)  117(11.9%)  169(17.2%)  179(18.3%)  174(17.8%)  259(26.4%) | 180(120-360)  180(120-390)  180(120-300)  180(120-300)  180(120-300)  240(180-360) | 7(8.5%)  17(14.5%)  20(11.8%)  16(8.9%)  11(6.3%)  27(10.4%) | 0.55(0.22-1.39)  1.00  0.79(0.39-1.58)  0.58(0.28-1.19)  0.40(0.18-0.88)*  0.68(0.36-1.31) | 0.41(0.14-1.14)  1.00  0.91(0.45-1.86)  0.87(0.41-1.84)  0.83(0.35-1.95)  1.43(0.66-3.09) | 0.54(0.18-1.64)  1.00  0.68(0.32-1.42)  0.74(0.34-1.61)  0.73(0.28-1.88)  1.13(0.39-3.22) |
| **Age stopped education**  Up to 15 years (ref)  16-19 years  20+ years  Still studying | 503(54.4%)  253(27.4%)  138(14.9%)  30(3.2%) | 180(120-300)  180(120-300)  240(165-420)  300(108.75-420) | 30(6%)  30(11.9%)  28(20.3%)  5(16.7%) | 1.00  2.12(1.25-3.61)*  4.01(2.30-6.99)**  3.15(1.13-8.82)* | 1.00  2.64(1.44-4.83)*  4.77(2.55-8.92)**  6.24(1.8-21.58)* |  |
| **Occupation**  Self-employed  Managers  Other white collars  Manual workers (ref)  House persons  Unemployed  Retired  Students | 62(6.3%)  70(7.1%)  64(6.5%)  228(23.3%)  77(7.9%)  146(14.9%)  303(30.9%)  30(3.1%) | 300(97.50-390)  300(180-420)  300(180-480)  180(75-240)  180(75-300)  180(120-240)  240(135-360)  300(108.75-420) | 11(17.7%)  16(22.9%)  19(29.7%)  8(3.5%)  1(1.3%)  8(5.5%)  30(9.9%)  5(16.7%) | 5.93(2.27-15.50)**  8.15(3.31-20.03)**  11.61(4.79-28.16)**  1.00  0.36(0.04-2.94)  1.59(0.58-4.35)  3.02(1.36-6.72)*  5.50(1.67-18.11)* |  | 5.34(2.01-14.15)*  7.83(3.14-19.51)**  12.30(4.95-30.56)**  1.00  0.35(0.04-2.98)  1.60(0.58-4.37)  2.22(0.76-6.48)  6.50(1.67-25.28)* |

*p<0.05 **p<0.001

S1 File, Table V. Sample characteristics and prevalence, univariate and multivariate odds ratio (OR) of sitting more than 7.5 hours per day, by gender, age, education and occupation, in the Republic of Cyprus. Because of co-linearity between the Education and Occupation variables we constructed two multivariate models: one including Education excluding Occupation (Model 1) and vice versa (Model 2).

| **Republic of Cyprus** | **N (% total population)** | **Median (25^th^-75^th^ percentile) sitting minutes per day** | **N (within group %) sitting >7.5 hours per day** | **Univariate OR (95% CI) of sitting >7.5 hours per day** | **Model 1: Multivariate^b^ OR (95% CI) of sitting >7.5 hours per day** | **Model 2: Multivariate^a^ OR (95% CI) of sitting >7.5 hours per day** |
| --- | --- | --- | --- | --- | --- | --- |
| **Overall** | 482(100%) | 300(180-420) | 92(19.1%) | - | - | - |
| **Gender**  Male (ref)  Female | 225(46.7%)  257(53.3%) | 300(180-420)  300(180-420) | 39(17.3%)  53(20.6%) | 1.00  1.24(0.78-1.96) | 1.00  1.27(0.79-2.04) | 1.00  1.29(0.76-2.18) |
| **Age**  18-24 years  25-34 years (ref)  35-44 years  45-54 years  55-64 years  65+ years | 33(6.8%)  84(17.4%)  79(16.4%)  76(15.8%)  84(17.4%)  126(26.1%) | 360(240-480)  240(180-420)  240(180-360)  300(180-420)  240(180-360)  300(240-420) | 9(27.3%)  17(20.2%)  13(16.5%)  17(22.4%)  7(8.3%)  29(23%) | 1.48(0.58-3.76)  1.00  0.78(0.35-1.72)  1.14(0.53-2.42)  0.36(0.14-0.92)*  1.18(0.60-2.31) | 1.61(0.51-5.06)  1.00  0.87(0.38-1.97)  1.27(0.58-2.76)  0.44(0.16-1.17)  1.38(0.65-2.96) | 1.86(0.54-6.41)  1.00  0.88(0.38-2.06)  1.13(0.50-2.54)  0.49(0.17-1.38)  1.61(0.54-4.80) |
| **Age stopped education**  Up to 15 years (ref)  16-19 years  20+ years  Still studying | 127(26.6%)  188(39.4%)  142(29.8%)  20(4.2%) | 300(180-360)  240(180-360)  300(180-420)  360(240-465) | 22(17.3%)  31(16.5%)  33(23.2%)  5(25%) | 1.00  0.94(0.52-1.72)  1.44(0.79-2.64)  1.59(0.52-4.83) | 1.00  1.01(0.52-1.94)  1.50(0.76-2.98)  1.09(0.25-4.78) |  |
| **Occupation**  Self-employed  Managers  Other white collars  Manual workers (ref)  House persons  Unemployed  Retired  Students | 35(7.3%)  35(7.3%)  65(13.5%)  74(15.4%)  68(14.1%)  65(13.5%)  120(24.9%)  20(4.1%) | 240(180-360)  300(165-420)  420(300-480)  180(120-300)  180(120-360)  300(210-420)  300(240-420)  360(240-465) | 5(14.3%)  7(20%)  27(41.5%)  6(8.1%)  8(11.8%)  8(12.3%)  26(21.7%)  5(25%) | 1.89(0.53-6.67)  2.83(0.87-9.18)  8.05(3.05-21.23)**  1.00  1.51(0.05-4.60)  1.59(0.52-4.85)  3.13(1.22-8.03)*  3.78(1.02-14.03)* |  | 1.92(0.52-7.05)  2.96(0.91-9.70)  7.37(2.77-19.64)**  1.00  1.21(0.35-4.16)  1.47(0.47-4.57)  2.23(0.62-8.03)  1.99(0.39-10.07) |

*p<0.05 **p<0.001

S1 File, Table W. Sample characteristics and prevalence, univariate and multivariate odds ratio (OR) of sitting more than 7.5 hours per day, by gender, age, education and occupation, in Romania. Because of co-linearity between the Education and Occupation variables we constructed two multivariate models: one including Education excluding Occupation (Model 1) and vice versa (Model 2).

| **Romania** | **N (% total population)** | **Median (25^th^-75^th^ percentile) sitting minutes per day** | **N (within group %) sitting >7.5 hours per day** | **Univariate OR (95% CI) of sitting >7.5 hours per day** | **Model 1: Multivariate^b^ OR (95% CI) of sitting >7.5 hours per day** | **Model 2: Multivariate^a^ OR (95% CI) of sitting >7.5 hours per day** |
| --- | --- | --- | --- | --- | --- | --- |
| **Overall** | 927(100%) | 240(120-360) | 133(14.3%) | - | - | - |
| **Gender**  Male (ref)  Female | 471(50.8%)  456(49.2%) | 240(120-360)  240(120-360) | 73(15.5%)  60(13.2%) | 1.00  0.83(0.57-1.19) | 1.00  0.89(0.61-1.31) | 1.00  0.82(0.55-1.21) |
| **Age**  18-24 years  25-34 years (ref)  35-44 years  45-54 years  55-64 years  65+ years | 72(7.8%)  127(13.7%)  171(18.4%)  153(16.5%)  217(23.4%)  187(20.2%) | 240(120-360)  180(120-360)  240(120-360)  180(120-360)  240(120-300)  300(180-420) | 11(15.3%)  19(15%)  25(14.6%)  23(15%)  20(9.2%)  35(18.7%) | 1.03(0.46-2.30)  1.00  0.97(0.51-1.86)  1.01(0.52-1.94)  0.58(0.30-1.13)  1.31(0.71-2.41) | 0.66(0.21-2.11)  1.00  1.22(0.62-2.39)  1.30(0.65-2.59)  0.80(0.40-1.61)  2.09(1.07-4.07)* | 0.72(0.23-2.23)  1.00  1.19(0.59-2.39)  1.20(0.59-2.46)  0.89(0.37-2.14)  2.30(0.84-6.32) |
| **Age stopped education**  Up to 15 years (ref)  16-19 years  20+ years  Still studying | 162(18.1%)  463(51.7%)  244(27.2%)  27(3%) | 240(120-300)  180(120-300)  240(180-420)  360(240-420) | 17(10.5%)  50(10.8%)  57(23.4%)  6(22.2%) | 1.00  1.03(0.58-1.85)  2.60(1.45-4.66)*  2.44(0.86-6.88) | 1.00  1.32(0.70-2.49)  3.32(1.75-6.29)**  5.57(1.24-24.94)* |  |
| **Occupation**  Self-employed  Managers  Other white collars  Manual workers (ref)  House persons  Unemployed  Retired  Students | 85(9.2%)  73(7.9%)  62(6.7%)  190(20.5%)  100(10.8%)  50(5.4%)  340(36.7%)  27(2.9%) | 180(120-360)  240(180-480)  360(240-480)  180(120-240)  180(75-300)  240(120-300)  240(180-360)  360(240-420) | 11(12.9%)  21(28.8%)  25(40.3%)  17(8.9%)  2(2%)  2(4%)  49(14.4%)  6(22.2%) | 1.51(0.68-3.39)  4.11(2.02-8.36)**  6.88(3.38-14.00)**  1.00  0.21(0.05-0.92)*  0.42(0.10-1.90)  1.71(0.96-3.07)  2.91(1.03-8.19)* |  | 1.43(0.63-3.22)  4.18(2.04-8.54)**  7.23(3.51-14.90)**  1.00  0.20(0.04-0.90)*  0.43(0.10-1.94)  1.17(0.48-2.87)  4.54(1.07-19.22)* |

*p<0.05 **p<0.001

S1 File, Table X. Sample characteristics and prevalence, univariate and multivariate odds ratio (OR) of sitting more than 7.5 hours per day, by gender, age, education and occupation, in Slovakia. Because of co-linearity between the Education and Occupation variables we constructed two multivariate models: one including Education excluding Occupation (Model 1) and vice versa (Model 2).

| **Slovakia** | **N (% total population)** | **Median (25^th^-75^th^ percentile) sitting minutes per day** | **N (within group %) sitting >7.5 hours per day** | **Univariate OR (95% CI) of sitting >7.5 hours per day** | **Model 1: Multivariate^b^ OR (95% CI) of sitting >7.5 hours per day** | **Model 2: Multivariate^a^ OR (95% CI) of sitting >7.5 hours per day** |
| --- | --- | --- | --- | --- | --- | --- |
| **Overall** | 956(100%) | 300(180-420) | 190(19.9%) | - | - | - |
| **Gender**  Male (ref)  Female | 405(42.4%)  551(57.6%) | 300(180-420)  300(180-420) | 78(19.3%)  112(20.3%) | 1.00  1.07(0.78-1.48) | 1.00  1.08(0.78-1.51) | 1.00  1.06(0.75-1.49) |
| **Age**  18-24 years  25-34 years (ref)  35-44 years  45-54 years  55-64 years  65+ years | 88(9.2%)  147(15.4%)  193(20.2%)  214(22.4%)  174(18.2%)  140(14.6%) | 360(255-480)  300(180-420)  300(180-420)  300(180-420)  300(180-360)  360(180-420) | 25(28.4%)  29(19.7%)  41(21.2%)  46(21.5%)  23(13.2%)  26(18.6%) | 1.62(0.87-2.99)  1.00  1.10(0.64-1.87)  1.11(0.66-1.88)  0.62(0.34-1.13)  0.93(0.52-1.67) | 1.65(0.70-3.87)  1.00  1.16(0.67-2.00)  1.20(0.71-2.05)  0.66(0.36-1.22)  0.98(0.53-1.81) | 2.24(0.88-5.68)  1.00  1.01(0.57-1.77)  1.03(0.59-1.80)  0.76(0.38-1.53)  1.32(0.53-3.30) |
| **Age stopped education**  Up to 15 years (ref)  16-19 years  20+ years  Still studying | 48(5.1%)  648(68.9%)  186(19.8%)  59(6.3%) | 300(180-360)  300(180-420)  360(240-480)  360(300-480) | 7(14.6%)  110(17%)  52(28%)  17(28.8%) | 1.00  1.20(0.52-2.74)  2.27(0.96-5.39)  2.37(0.89-6.32) | 1.00  1.07(0.46-2.51)  2.04(0.84-4.94)  1.37(0.40-4.72) |  |
| **Occupation**  Self-employed  Managers  Other white collars  Manual workers (ref)  House persons  Unemployed  Retired  Students | 55(5.8%)  67(7%)  188(19.7%)  271(28.3%)  20(2.1%)  58(6.1%)  238(24.9%)  59(6.2%) | 300(180-420)  360(240-480)  360(240-480)  240(180-360)  240(135-345)  240(180-360)  300(225-420)  360(300-480) | 10(18.2%)  24(35.8%)  68(36.2%)  28(10.3%)  1(5%)  4(6.9%)  38(16%)  17(28.8%) | 1.93(0.88-4.25)  4.84(2.57-9.14)**  4.92(3.01-8.04)**  1.00  0.46(0.06-3.54)  0.64(0.22-1.91)  1.65(0.98-2.78)  3.51(1.77-6.97)** |  | 1.97(0.89-4.35)  4.93(2.60-9.35)**  4.92(3-80.08)**  1.00  0.41(0.05-3.22)  0.57(0.19-1.74)  1.54(0.70-3.38)  1.66(0.59-4.67) |

*p<0.05 **p<0.001

S1 File, Table Y. Sample characteristics and prevalence, univariate and multivariate odds ratio (OR) of sitting more than 7.5 hours per day, by gender, age, education and occupation, in Slovenia. Because of co-linearity between the Education and Occupation variables we constructed two multivariate models: one including Education excluding Occupation (Model 1) and vice versa (Model 2).

| **Slovenia** | **N (% total population)** | **Median (25^th^-75^th^ percentile) sitting minutes per day** | **N (within group %) sitting >7.5 hours per day** | **Univariate OR (95% CI) of sitting >7.5 hours per day** | **Model 1: Multivariate^b^ OR (95% CI) of sitting >7.5 hours per day** | **Model 2: Multivariate^a^ OR (95% CI) of sitting >7.5 hours per day** |
| --- | --- | --- | --- | --- | --- | --- |
| **Overall** | 1094(100%) | 240(120-300) | 134(12.2%) | - | - | - |
| **Gender**  Male (ref)  Female | 455(41.6%)  639(58.4%) | 240(120-300)  180(120-300) | 64(14.1%)  70(11%) | 1.00  0.75(0.52-1.08) | 1.00  0.79(0.54-1.15) | 1.00  0.79(0.54-1.16) |
| **Age**  18-24 years  25-34 years (ref)  35-44 years  45-54 years  55-64 years  65+ years | 76(6.9%)  167(15.3%)  148(13.5%)  189(17.3%)  245(22.4%)  269(24.6%) | 300(180-420)  240(180-360)  180(120-300)  240(120-360)  180(120-300)  240(180-300) | 17(22.4%)  30(18%)  18(12.2%)  27(14.3%)  22(9%)  20(7.4%) | 1.32(0.67-2.57)  1.00  0.63(0.34-1.19)  0.76(0.43-1.34)  0.45(0.25-0.81)*  0.37(0.20-0.67)* | 1.27(0.56-2.86)  1.00  0.73(0.38-1.39)  0.84(0.47-1.51)  0.46(0.25-0.86)*  0.43(0.23-0.80)* | 1.63(0.68-3.91)  1.00  0.52(0.27-1.01)  0.69(0.38-1.26)  0.89(0.44-1.81)  1.23(0.50-3.03) |
| **Age stopped education**  Up to 15 years (ref)  16-19 years  20+ years  Still studying | 182(17%)  496(46.2%)  329(30.7%)  66(6.2%) | 180(120-300)  180(120-300)  240(180-360)  300(180-420) | 14(7.7%)  46(9.3%)  57(17.3%)  15(22.7%) | 1.00  1.23(0.66-2.29)  2.52(1.36-4.65)*  3.53(1.60-7.80)* | 1.00  0.92(0.48-1.78)  1.90(1.00-3.61)  1.54(0.56-4.19) |  |
| **Occupation**  Self-employed  Managers  Other white collars  Manual workers (ref)  House persons  Unemployed  Retired  Students | 84(7.7%)  89(8.1%)  105(9.6%)  180(16.5%)  29(2.7%)  103(9.4%)  438(40%)  66(6%) | 180(120-300)  240(180-360)  360(180-480)  180(120-300)  120(75-300)  180(120-300)  180(120-300)  300(180-420) | 16(19%)  18(20.2%)  33(31.4%)  19(10.6%)  2(6.9%)  5(4.9%)  26(5.9%)  15(22.7%) | 1.99(0.97-4.11)  2.15(1.06-4.34)*  3.88(2.07-7.29)**  1.00  0.63(0.14-2.85)  0.43(0.16-1.20)  0.54(0.29-0.99)*  2.49(1.18-5.26)* |  | 2.19(1.05-4.58)*  2.38(1.16-4.86)*  4.43(2.32-8.45)**  1.00  0.63(0.13-2.95)  0.41(0.15-1.15)  0.42(0.18-1.00)  1.48(0.58-3.81) |

*p<0.05 **p<0.001

S1 File, Table Z. Sample characteristics and prevalence, univariate and multivariate odds ratio (OR) of sitting more than 7.5 hours per day, by gender, age, education and occupation, in Spain. Because of co-linearity between the Education and Occupation variables we constructed two multivariate models: one including Education excluding Occupation (Model 1) and vice versa (Model 2).

| **Spain** | **N (% total population)** | **Median (25^th^-75^th^ percentile) sitting minutes per day** | **N (within group %) sitting >7.5 hours per day** | **Univariate OR (95% CI) of sitting >7.5 hours per day** | **Model 1: Multivariate^b^ OR (95% CI) of sitting >7.5 hours per day** | **Model 2: Multivariate^a^ OR (95% CI) of sitting >7.5 hours per day** |
| --- | --- | --- | --- | --- | --- | --- |
| **Overall** | 982(100%) | 240(180-360) | 87(8.9%) | - | - | - |
| **Gender**  Male (ref)  Female | 462(47%)  520(53%) | 240(180-360)  240(180-300) | 50(10.8%)  37(7.1%) | 1.00  0.63(0.41-0.99)* | 1.00  0.59(0.37-0.95)* | 1.00  0.83(0.51-1.35) |
| **Age**  18-24 years  25-34 years (ref)  35-44 years  45-54 years  55-64 years  65+ years | 94(9.6%)  159(16.2%)  207(21.1%)  184(18.7%)  137(14%)  201(20.5%) | 300(180-360)  240(180-300)  240(180-360)  240(120-300)  240(180-300)  270(240-360) | 9(9.6%)  15(9.4%)  24(11.6%)  13(7.1%)  8(5.8%)  18(9%) | 1.02(0.43-2.42)  1.00  1.26(0.64-2.49)  0.73(0.34-1.58)  0.60(0.24-1.45)  0.94(0.46-1.94) | 0.47(0.15-1.42)  1.00  1.90(0.90-3.99)  1.20(0.52-2.78)  1.14(0.43-3.03)  1.71(0.69-4.24) | 0.49(0.16-1.51)  1.00  1.70(0.78-3.72)  0.98(0.41-2.35)  0.79(0.27-2.29)  1.20(0.37-3.92) |
| **Age stopped education**  Up to 15 years (ref)  16-19 years  20+ years  Still studying | 372(38.6%)  307(31.8%)  224(23.2%)  61(6.3%) | 240(180-300)  240(180-300)  240(180-360)  360(240-420) | 21(5.6%)  15(4.9%)  34(15.2%)  13(21.3%) | 1.00  0.86(0.44-1.70)  2.99(1.69-5.30)**  4.53(2.13-9.63)** | 1.00  0.94(0.45-1.96)  3.14(1.63-6.05)*  10.58(3.29-34.03)** |  |
| **Occupation**  Self-employed  Managers  Other white collars  Manual workers (ref)  House persons  Unemployed  Retired  Students | 57(5.8%)  48(4.9%)  65(6.6%)  216(22%)  126(12.8%)  206(21%)  203(20.7%)  61(6.2%) | 240(135-300)  300(180-420)  360(240-480)  180(120-300)  240(180-300)  240(180-300)  300(240-360)  360(240-420) | 7(12.3%)  11(22.9%)  21(32.3%)  6(2.8%)  3(2.4%)  6(2.9%)  20(9.9%)  13(21.3%) | 4.90(1.58-15.22)*  10.41(3.63-29.86)**  16.71(6.37-43.79)**  1.00  0.85(0.21-3.47)  1.05(0.33-3.31)  3.83(1.50-9.73)*  9.48(3.43-26.21)** |  | 5.14(1.64-16.10)*  10.45(3.62-30.19)**  16.00(6.07-42.19)**  1.00  1.03(0.24-4.52)  1.07(0.34-3.37)  3.92(1.14-13.54)*  17.87(4.92-64.94)** |

*p<0.05 **p<0.001

S1 File, Table AA. Sample characteristics and prevalence, univariate and multivariate odds ratio (OR) of sitting more than 7.5 hours per day, by gender, age, education and occupation, in Sweden. Because of co-linearity between the Education and Occupation variables we constructed two multivariate models: one including Education excluding Occupation (Model 1) and vice versa (Model 2).

| **Sweden** | **N (% total population)** | **Median (25^th^-75^th^ percentile) sitting minutes per day** | **N (within group %) sitting >7.5 hours per day** | **Univariate OR (95% CI) of sitting >7.5 hours per day** | **Model 1: Multivariate^b^ OR (95% CI) of sitting >7.5 hours per day** | **Model 2: Multivariate^a^ OR (95% CI) of sitting >7.5 hours per day** |
| --- | --- | --- | --- | --- | --- | --- |
| **Overall** | 974(100%) | 300(240-420) | 236(24.2%) | - | - | - |
| **Gender**  Male (ref)  Female | 497(51%)  477(49%) | 360(240-480)  300(240-420) | 130(26.2%)  106(22.2%) | 1.00  0.81(0.60-1.08) | 1.00  0.81(0.60-1.11) | 1.00  0.86(0.62-1.17) |
| **Age**  18-24 years  25-34 years (ref)  35-44 years  45-54 years  55-64 years  65+ years | 23(2.4%)  46(4.7%)  149(15.3%)  157(16.1%)  210(21.6%)  389(39.9%) | 450(345-540)  360(240-540)  360(300-480)  360(240-480)  360(240-480)  300(180-360) | 12(52.2%)  18(39.1%)  48(32.2%)  44(28%)  61(29%)  53(13.6%) | 1.70(0.62-4.66)  1.00  0.74(0.37-1.47)  0.61(0.30-1.20)  0.64(0.33-1.24)  0.25(0.13-0.47)* | 0.55(0.15-2.04)  1.00  0.81(0.40-1.64)  0.66(0.32-1.34)  0.73(0.37-1.46)  0.29(0.14-0.57)** | 1.03(0.28-3.80)  1.00  0.73(0.35-1.50)  0.69(0.34-1.42)  0.86(0.42-1.75)  0.57(0.25-1.32) |
| **Age stopped education**  Up to 15 years (ref)  16-19 years  20+ years  Still studying | 68(7%)  217(22.5%)  654(67.8%)  26(2.7%) | 300(180-420)  300(240-420)  360(240-420)  480(405-540) | 11(16.2%)  46(21.2%)  160(24.5%)  17(65.4%) | 1.00  1.39(0.68-2.87)  1.68(0.86-3.28)  9.79(3.48-27.53)** | 1.00  0.89(0.42-1.89)  1.05(0.52-2.13)  5.31(1.43-19.76)* |  |
| **Occupation**  Self-employed  Managers  Other white collars  Manual workers (ref)  House persons  Unemployed  Retired  Students | 66(6.8%)  204(20.9%)  148(15.2%)  115(11.8%)  3(0.3%)  25(2.6%)  387(39.7%)  26(2.7%) | 360(240-420)  360(300-540)  420(300-540)  300(180-390)  360(300-420)  300(240-360)  300(180-360)  480(405-540) | 12(18.2%)  75(36.8%)  61(41.2%)  17(14.8%)  0(0%)  4(16%)  50(12.9%)  17(65.4%) | 1.28(0.57-2.88)  3.35(1.86-6.04)**  4.04(2.20-7.44)**  1.00  0(0-)  1.10(0.34-3.60)  0.86(0.47-1.55)  10.89(4.18-28.38)** |  | 1.34(0.59-3.05)  3.38(1.86-6.12)**  4.11(2.22-7.61)**  1.00  0(0-)  1.01(0.30-3.34)  1.12(0.53-2.36)  9.24(2.92-29.26)** |

*p<0.05 **p<0.001

S1 File, Table BB. Sample characteristics and prevalence, univariate and multivariate odds ratio (OR) of sitting more than 7.5 hours per day, by gender, age, education and occupation, in the United Kingdom. Because of co-linearity between the Education and Occupation variables we constructed two multivariate models: one including Education excluding Occupation (Model 1) and vice versa (Model 2).

| **United Kingdom** | **N (% total population)** | **Median (25^th^-75^th^ percentile) sitting minutes per day** | **N (within group %) sitting >7.5 hours per day** | **Univariate OR (95% CI) of sitting >7.5 hours per day** | **Model 1: Multivariate^b^ OR (95% CI) of sitting >7.5 hours per day** | **Model 2: Multivariate^a^ OR (95% CI) of sitting >7.5 hours per day** |
| --- | --- | --- | --- | --- | --- | --- |
| **Overall** | 1267(100%) | 300(180-420) | 244(19.3%) | - | - | - |
| **Gender**  Male (ref)  Female | 593(46.8%)  674(53.2%) | 300(180-420)  300(180-360) | 133(22.4%)  111(16.5%) | 1.00  0.68(0.52-0.90)* | 1.00  0.70(0.52-0.92)* | 1.00  0.70(0.52-0.94)* |
| **Age**  18-24 years  25-34 years (ref)  35-44 years  45-54 years  55-64 years  65+ years | 105(8.3%)  197(15.5%)  168(13.3%)  166(13.1%)  186(14.7%)  445(35.1%) | 240(180-300)  240(120-360)  240(120-420)  300(180-420)  300(195-420)  300(240-420) | 10(9.5%)  37(18.8%)  34(20.2%)  35(21.1%)  42(22.6%)  86(19.3%) | 0.46(0.22-0.96)*  1.00  1.10(0.65-1.84)  1.16(0.69-1.94)  1.26(0.77-2.07)  1.04(0.68-1.59) | 0.41(0.18-0.92)*  1.00  1.09(0.64-1.84)  1.25(0.74-2.10)  1.26(0.75-2.09)  0.95(0.59-1.53) | 0.44(0.19-1.03)  1.00  0.96(0.56-1.67)  1.00(0.58-1.73)  1.02(0.57-1.81)  0.57(0.31-1.06) |
| **Age stopped education**  Up to 15 years (ref)  16-19 years  20+ years  Still studying | 315(25%)  606(48.1%)  292(23.2%)  48(3.8%) | 300(180-420)  300(180-360)  300(180-420)  300(195-420) | 71(22.5%)  98(16.2%)  65(22.3%)  9(18.8%) | 1.00  0.66(0.47-0.93)*  0.98(0.67-1.44)  0.79(0.37-1.72) | 1.00  0.67(0.45-0.99)*  0.94(0.61-1.46)  1.39(0.55-3.48) |  |
| **Occupation**  Self-employed  Managers  Other white collars  Manual workers (ref)  House persons  Unemployed  Retired  Students | 73(5.8%)  133(10.5%)  106(8.4%)  230(18.2%)  97(7.7%)  78(6.2%)  502(39.6%)  48(3.8%) | 240(120-330)  360(180-480)  420(300-480)  210(120-300)  240(180-300)  240(120-360)  300(240-420)  300(195-420) | 10(13.7%)  41(30.8%)  37(34.9%)  18(7.8%)  8(8.2%)  11(14.1%)  110(21.9%)  9(18.8%) | 1.87(0.82-4.26)  5.25(2.86-9.62)**  6.32(3.38-11.8)**  1.00  1.06(0.44-2.52)  1.93(0.87-4.30)  3.30(1.95-5.59)**  2.72(1.14-6.49)* |  | 1.69(0.73-3.89)  5.01(2.71-9.26)**  6.65(3.54-12.52)**  1.00  1.32(0.54-3.20)  1.95(0.87-4.36)  4.63(2.41-8.91)**  4.24(1.61-11.16)* |

*p<0.05 **p<0.001
