# Supplementary material for: European Sitting Championship: Prevalence and Correlates of Self-Reported Sitting Time in the 28 European Union Member States
Source: PLoS One. 2016 Mar 2;11(3):e0149320. doi: 10.1371/journal.pone.0149320 (PMC4774909; doi:10.1371/journal.pone.0149320)
Supplement: S1 Table — The countries are ordered based on the univariate OR. Because of co-linearity between the Education and Occupation variables we constructed two multivariate models: one including Education excluding Occupation (Model 1) and vice versa (Model 2). (DOCX) [file pone.0149320.s002.docx]

S1 Table. Sample characteristics and prevalence, univariate and multivariate odds ratio (OR) of sitting more than 7.5 hours per day, by country and socio-demographic characteristics. The countries are ordered based on the univariate OR. Because of co-linearity between the Education and Occupation variables we constructed two multivariate models: one including Education excluding Occupation (Model 1) and vice versa (Model 2).

|  | **N (% total population)** | **Median (25^th^-75^th^ percentile) sitting minutes per day** | **N (within group %) sitting >7.5 hours per day** | **Univariate OR (95% CI) of sitting >7.5 hours per day** | **Model 1: Multivariate^b^ OR (95% CI) of sitting >7.5 hours per day** | **Model 2: Multivariate^a^ OR (95% CI) of sitting >7.5 hours per day** |
| --- | --- | --- | --- | --- | --- | --- |
| **Overall** | 26617 (100%) | 300 (180-420) | 4924 (18.5%) | - | - | - |
| **Country**  **(ref: all other countries)**  Netherlands  Denmark  Czech Republic  Sweden  Estonia  Croatia  Finland  Luxembourg  Greece  Slovakia  Bulgaria  United Kingdom  Republic of Cyprus  Austria  Germany  France  Poland  Belgium  Latvia  Lithuania  Romania  Slovenia  Malta  Hungary  Italy  Ireland  Portugal  Spain | 991 (3.7%)  984 (3.7%)  985 (3.7%)  974 (3.7%)  983 (3.7%)  978 (3.7%)  942 (3.5%)  484 (1.8%)  973 (3.7%)  956 (3.6%)  947 (3.6%)  1267 (4.8%)  482 (1.8%)  948 (3.6%)  1531 (5.8%)  991 (3.7%)  879 (3.3%)  1042 (3.9%)  964 (3.6%)  963 (3.6%)  927 (3.5%)  1094 (4.1%)  488 (1.8%)  974 (3.7%)  955 (3.6%)  953 (3.6%)  980 (3.7%)  982 (3.7%) | 360 (240-480)  360 (240-480)  300 (180-480)  300 (240-420)  300 (180-420)  300 (180-420)  300 (240-420)  300 (180-420)  300 (180-420)  300 (180-420)  300 (240-420)  300 (180-420)  300 (180-420)  300 (240-420)  300 (180-420)  240 (180-420)  240 (180-360)  300 (180-420)  300 (180-420)  300 (180-420)  240 (120-360)  240 (120-300)  240 (120-360)  240 (120-360)  240 (180-360)  240 (180-360)  180 (120-360)  240 (180-360) | 318 (32.1%)  312 (31.7%)  261 (26.5%)  236 (24.2%)  224 (22.8%)  222 (22.7%)  201 (21.3%)  100 (20.7%)  194 (19.9%)  190 (19.9%)  187 (19.7%)  244 (19.3%)  92 (19.1%)  180 (19.0%)  280 (18.3%)  181 (18.3%)  158 (18.0%)  186 (17.9%)  168 (17.4%)  161 (16.7%)  133 (14.3%)  134 (12.2%)  58 (11.9%)  115 (11.8%)  105 (11.0%)  99 (10.4%)  98 (10.0%)  87 (8.9%) | 2.16 (1.88-2.47)**  2.12 (1.84-2.43)**  1.62 (1.40-1.87)**  1.43 (1.23-1.66)**  1.32 (1.13-1.53)**  1.31 (1.12-1.52)*  1.20 (1.03-1.41)*  1.15 (0.92-1.44)  1.10 (0.94-1.29)  1.10 (0.93-1.29)  1.09 (0.92-1.28)  1.05 (0.91-1.22)  1.04 (0.83-1.31)  1.03 (0.88-1.22)  0.99 (0.86-1.13)  0.98 (0.84-1.16)  0.96 (0.81-1.15)  0.96 (0.81-1.12)  0.93 (0.78-1.10)  0.88 (0.74-1.05)  0.73 (0.61-0.88)*  0.60 (0.50-0.73)**  0.59 (0.45-0.78)**  0.58 (0.48-0.71)**  0.53 (0.44-0.66)**  0.50 (0.41-0.62)**  0.48 (0.39-0.59)**  0.42 (0.34-0.52)** | 1.94 (1.69-2.23)**  1.68 (1.46-1.94)**  1.79 (1.54-2.08)**  1.17 (1.00-1.36)  1.21 (1.04-1.42)*  1.35 (1.16-1.59)**  1.06 (0.90-1.25)  1.09 (0.87-1.37)  1.11 (0.94-1.31)  1.15 (0.98-1.36)  1.13 (0.96-1.33)  1.13 (0.98-1.31)  1.07 (0.85-1.36)  1.18 (1.00-1.40)  1.01 (0.88-1.15)  0.96 (0.81-1.13)  0.95 (0.80-1.14)  0.90 (0.76-1.06)  0.91 (0.77-1.08)  0.83 (0.69-0.99)*  0.78 (0.64-0.94)*  0.60 (0.50-0.73)**  0.67 (0.51-0.90)*  0.67 (0.55-0.82)**  0.57 (0.46-0.70)**  0.51 (0.41-0.63)**  0.56 (0.45-0.70)**  0.43 (0.34-0.53)** | 2.02 (1.75-2.33)**  2.00 (1.73-2.30)**  1.36 (1.17-1.58)**  1.11 (0.95-1.30)  1.23 (1.05-1.44)*  1.32 (1.13-1.55)*  1.18 (1.00-1.39)*  1.19 (0.94-1.49)  1.18 (1.00-1.39)  1.04 (0.88-1.23)  1.10 (0.93-1.30)  1.07 (0.93-1.25)  1.10 (0.86-1.39)  0.94 (0.79-1.12)  0.97 (0.84-1.11)  1.06 (0.89-1.25)  0.99 (0.83-1.19)  0.91 (0.77-1.08)  0.98 (0.83-1.17)  0.90 (0.75-1.07)  0.83 (0.68-1.00)*  0.59 (0.49-0.71)**  0.74 (0.56-0.99)*  0.61 (0.50-0.75)**  0.49 (0.39-0.60)**  0.56 (0.45-0.70)**  0.55 (0.44-0.68)**  0.50 (0.40-0.63)** |
| **Gender**  Male (ref)  Female | 12062 (45.3%)  14555 (54.7%) | 300 (180-420)  300 (180-420) | 2357 (19.5%)  2567 (17.6%) | 1.00  0.88 (0.83-0.94)** | 1.00  0.88 (0.83-0.94)** | 1.00  0.90 (0.85-0.97)* |
| **Age**  18-24 years  25-34 years (ref)  35-44 years  45-54 years  55-64 years  65+ years | 2231 (8.4%)  3852 (14.5%)  4455 (16.7%)  4786 (18.0%)  4887 (18.4%)  6406 (24.1%) | 300 (180-420)  240 (180-420)  240 (180-420)  300 (180-420)  300 (180-360)  300 (240-420) | 459 (20.6%)  736 (19.1%)  843 (18.9%)  898 (18.8%)  807 (16.5%)  1181 (18.4%) | 1.10 (0.96-1.25)  1.00  0.99 (0.89-1.10)  0.98 (0.88-1.09)  0.84 (0.75-0.94)*  0.96 (0.86-1.06) | 0.79 (0.67-0.93)*  1.00  1.05 (0.94-1.18)  1.05 (0.94-1.18)  0.89 (0.80-1.00)  1.01 (0.90-1.12) | 0.89 (0.76-1.06)  1.00  1.00 (0.89-1.13)  1.02 (0.91-1.14)  0.94 (0.83-1.06)  1.13 (0.97-1.31) |
| **Age when stopped education**  Up to 15 years (ref)  16-19 years  20+ years  Still studying | 4540 (17.4%)  11794 (45.2%)  8500 (32.6%)  1270 (4.9%) | 240 (180-360)  240 (180-360)  300 (180-420)  360 (300-480) | 629 (13.9%)  1830 (15.5%)  2006 (23.6%)  356 (28.0%) | 1.00  1.14 (1.04-1.26)*  1.92 (1.74-2.12)**  2.42 (2.09-2.81)** | 1.00  0.97 (0.87-1.08)  1.52 (1.36-1.70)**  2.53 (2.07-3.08)** |  |
| **Current occupation**  Self-employed  Managers  Other white collars  Manual workers (ref)  House persons  Unemployed  Retired  Students | 1971 (7.4%)  2662 (10.0%)  3205 (12.0%)  5368 (20.2%)  1929 (7.2%)  2153 (8.1%)  8059 (30.3%)  1270 (4.8%) | 240 (180-360)  360 (240-480)  360 (240-480)  240 (120-300)  240 (120-300)  240 (180-360)  300 (180-420)  360 (300-480) | 346 (17.6%)  806 (30.3%)  1125 (35.1%)  481 (9.0%)  129 (6.7%)  243 (11.3%)  1438 (17.8%)  356 (28.0%) | 2.16 (1.86-2.51)**  4.41 (3.89-5.00)**  5.50 (4.88-6.19)**  1.00  0.73 (0.60-0.89)*  1.29 (1.10-1.52)*  2.21 (1.98-2.46)**  3.96 (3.39-4.62)** |  | 2.08 (1.78-2.42)**  4.14 (3.65-4.70)**  5.44 (4.82-6.14)**  1.00  0.84 (0.68-1.03)  1.38 (1.17-1.63)**  1.96 (1.70-2.27)**  4.31 (3.54-5.24)** |

*p<0.05 **p<0.001. a. Adjusted for country, gender, age and age stopped education. b. Adjusted for country, gender, age and occupation.
